# Shielding Protection by Mesoporous Catalysts for Improving Plasma-Catalytic Ambient Ammonia Synthesis

**DOI:** 10.1021/jacs.2c01950

**Published:** 2022-06-22

**Authors:** Yaolin Wang, Wenjie Yang, Shanshan Xu, Shufang Zhao, Guoxing Chen, Anke Weidenkaff, Christopher Hardacre, Xiaolei Fan, Jun Huang, Xin Tu

**Affiliations:** † Department of Electrical Engineering and Electronics, University of Liverpool, Liverpool L69 3GJ, U.K.; ‡ School of Chemical and Biomolecular Engineering, Sydney Nano Institute, The University of Sydney, Sydney, New South Wales 2037, Australia; § Department of Chemical Engineering, School of Engineering, The University of Manchester, Oxford Road, Manchester M13 9PL, U.K.; ∥ Fraunhofer Research Institution for Materials Recycling and Resource Strategies IWKS, Brentanostraße 2a, Alzenau 63755, Germany; ⊥ Department of Materials and Earth Sciences, Materials and Resources, Technical University of Darmstadt, Alarich-Weiss-Str. 2, Darmstadt 64287, Germany

## Abstract

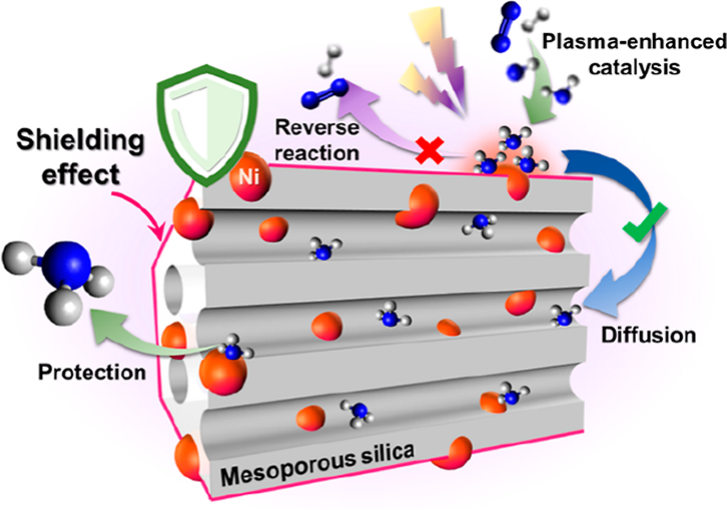

Plasma catalysis is a promising technology for decentralized small-scale ammonia (NH_3_) synthesis under mild conditions using renewable energy, and it shows great potential as an alternative to the conventional Haber–Bosch process. To date, this emerging process still suffers from a low NH_3_ yield due to a lack of knowledge in the design of highly efficient catalysts and the in situ plasma-induced reverse reaction (i.e., NH_3_ decomposition). Here, we demonstrate that a bespoke design of supported Ni catalysts using mesoporous MCM-41 could enable efficient plasma-catalytic NH_3_ production at 35 °C and 1 bar with >5% NH_3_ yield at 60 kJ/L. Specifically, the Ni active sites were deliberately deposited on the external surface of MCM-41 to enhance plasma–catalyst interactions and thus NH_3_ production. The desorbed NH_3_ could then diffuse into the ordered mesopores of MCM-41 to be shielded from decomposition due to the absence of plasma discharge in the mesopores of MCM-41, that is, “shielding protection”, thus driving the reaction forward effectively. This promising strategy sheds light on the importance of a rational design of catalysts specifically for improving plasma-catalytic processes.

## Introduction

Ammonia (NH_3_) is an important building block for fertilizers and many other chemicals. It is also a flexible long-term energy carrier and zero-carbon fuel.^1^ Today, ammonia is mainly produced from N_2_ and H_2_ on a large scale using the Haber–Bosch (H–B) process, which typically operates at high temperatures (650–750 K) and pressures (50–200 bar) in the presence of an Fe-based catalyst.^2,3^ This well-developed and centralized ammonia synthesis process (including the energy for hydrogen production) is energy-intensive, consuming 1–2% of the global energy supply and contributing to 1.44% of CO_2_ emissions and is only economically feasible on a large scale.^4^ Furthermore, large-scale centralized H–B facilities will face challenges in rapidly scaling production output to match the fluctuating input of renewable energy sources such as wind and solar power. Hence, there is growing interest in developing green and sustainable alternative technologies for decentralized renewable ammonia production under mild conditions.^5−10^

Non-thermal plasma (NTP) catalysis, also known as plasma catalysis, is an attractive hybrid technology for the activation of inert molecules (e.g., CO_2_, CH_4_, and N_2_) with strong chemical bonds at low temperatures and ambient pressure.^11−13^ In NTPs (such as the dielectric barrier discharge, DBD), electrons are highly energetic with an electron temperature of 10^4^–10^5^ K (1–10 eV), while the bulk gas temperature can remain as low as room temperature.^14^ Unlike thermal catalysis, which requires high temperatures to break the N≡N triple bond even on a catalyst surface, plasma activation can remove the equilibrium limitations experienced by thermal systems and enable conversions under milder bulk conditions,^15^ making the hybrid system effective for many challenging catalytic reactions.^16−19^ Previous studies have demonstrated that the plasma-induced vibrationally excited N_2_ can effectively lower the dissociative adsorption energy barrier of N_2_ molecules on Ni and Co nanoparticles (NPs), which are not active in thermal catalysis, allowing catalytic NH_3_ synthesis over Ni and Co NPs under mild plasma conditions.^20^ In addition, plasma-based module processes can be switched on and off instantly, offering great flexibility for decentralized small-scale ammonia synthesis using intermittent renewables, which can improve the economic competitiveness of the plasma process.

Although effective and promising, NH_3_ synthesis using the hybrid plasma-catalytic process has intrinsic challenges, such as the plasma-induced reverse reaction (NH_3_ decomposition)^21^ and the complexity of plasma–catalyst interactions. In the hybrid system, the desorbed ammonia from the catalyst surface can be easily decomposed in the plasma discharge via electron impact dissociation,^22^ which limits the practical ammonia yield that can be achieved as well as the energy yield for ammonia production. As such, this is regarded as one of the main obstacles in achieving a competitive NH_3_ synthesis rate when compared to the conventional H–B process. In non-catalytic systems, inert porous packing (such as zeolite 5A and 4A^23^) was recently found to be beneficial in improving NH_3_ synthesis rates, most likely due to in situ NH_3_ adsorption by the porous packing.^22^ Furthermore, the supported metal catalysts on mesoporous materials (e.g., Ru/MCM-41^24^ and Ni–Mg/SBA-15^25^) were also found to be very effective in promoting plasma-catalytic NH_3_ synthesis. Despite the fact that using porous materials or catalysts in plasma discharge improves the NH_3_ yield, the mechanism underlying such hybrid systems remains unclear. Moreover, due to the complex interactions between the plasma discharge and the catalyst in plasma-catalytic systems, rational design of catalysts that considers the new dimensions introduced by plasma discharges for highly effective plasma-catalytic NH_3_ synthesis has not yet been attempted.

Here, we propose a “shielding protection” catalyst design strategy based on the interaction between the plasma and a bespoke mesoporous catalyst to limit the plasma-induced ammonia decomposition during the plasma-catalytic ammonia synthesis in a DBD reactor. Specifically, we designed Ni supported on ordered mesoporous MCM-41 catalysts, that is, Ni/MCM-41, using a controlled deposition of Ni NPs on the MCM-41 support. The Ni NPs dispersed over the external surface of MCM-41 could significantly benefit NH_3_ production in the plasma-catalytic process due to their high accessibility. More importantly, the gradient of NH_3_ concentration across the mesoporous framework enabled the formed NH_3_ to diffuse into the mesopores, thus limiting the plasma-induced reverse reaction (NH_3_ decomposition) due to the absence of plasma discharge in the mesopores, that is, “shielding protection”, shifting the reaction equilibrium towards greater NH_3_ production. The rationally designed catalyst enabled high NH_3_ yields of >5% under plasma conditions at 60 kJ/L, which is among the best of the state-of-the-art plasma-assisted NH_3_ synthesis systems. Furthermore, comprehensive catalyst characterization and in situ characterization of plasma-catalyzed surfaces were carried out to elucidate the relevant mechanisms in the plasma-catalytic ammonia synthesis over the developed mesoporous catalysts.

## Results and Discussion

### Interaction of the Supported Ni Species with Plasma and Shielding Effect of Mesoporous Ni/MCM-41

**Figure 1:**
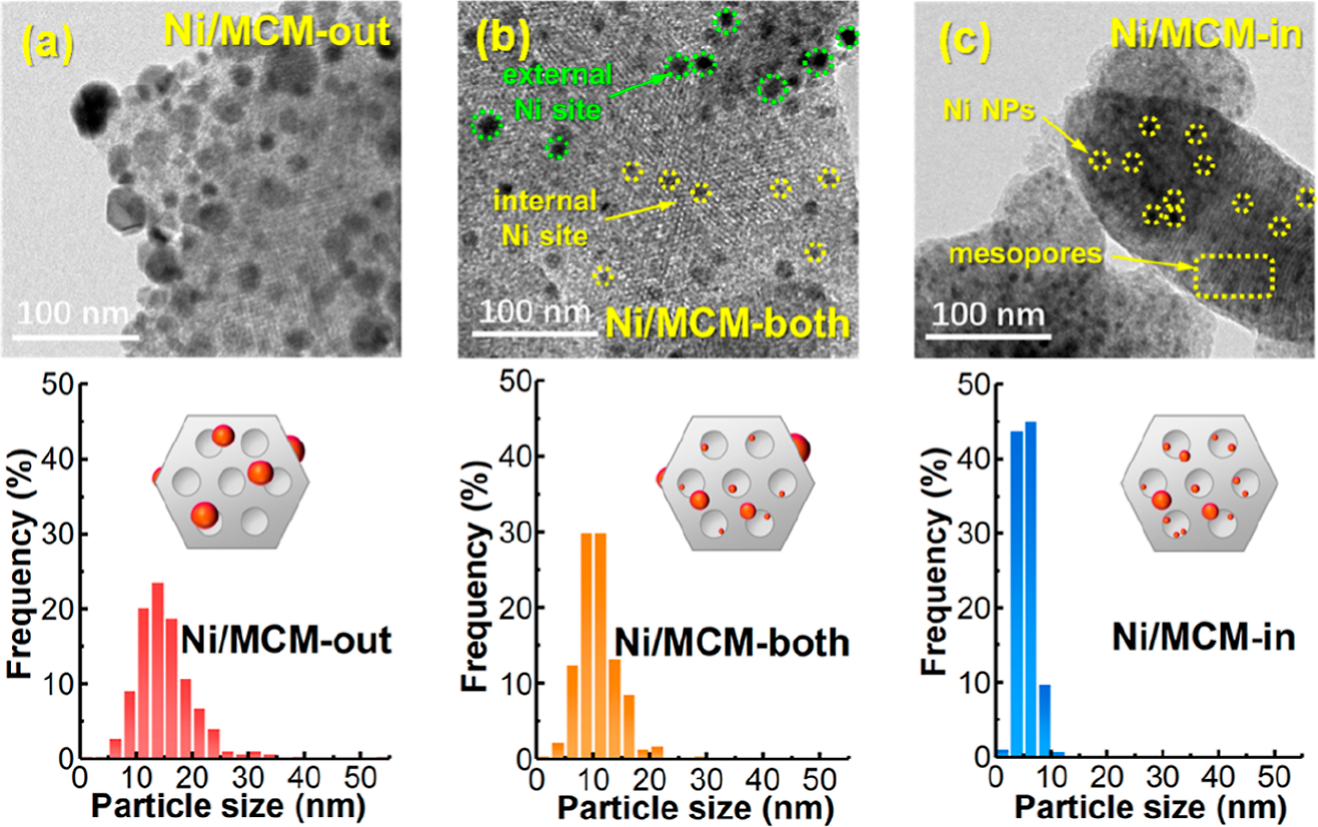
HRTEM image and PSD of (a) Ni/MCM-out, (b) Ni/MCM-both, and (c) Ni/MCM-in (all in the reduced state).

Based on the synthesis protocols,^26,27^ Ni NPs could be deposited in different locations of MCM-41, including exclusively within its mesoporous framework (Ni/MCM-in), mainly on its external surface (Ni/MCM-out), and across its framework (Ni/MCM-both). Table S1 lists the states of the catalysts during preparation as well as the corresponding characterization. High-resolution transmission electron microscopy (HRTEM) images (Figures 1a–c and S1) show the morphologies of the investigated catalysts, revealing the locations of Ni NPs in the different catalysts. In detail, the Ni NPs of Ni/MCM-out (Figure 1a) are mainly dispersed on the external surface of the support, with a wider distribution of Ni NPs and a larger mean particle size of ∼15 nm (compared to Ni/MCM-both and Ni/MCM-in, Figures 1b,c, relevant characterization data of the catalysts are shown in Figure S2 and Table S2 in the Supporting Information). Conversely, for Ni/MCM-in (Figure 1c), the Ni NPs are primarily confined to the mesopores of MCM-41, with a much narrower particle size distribution (PSD) centered at ∼5 nm due to the confinement effect of mesopores.

**Figure 2:**
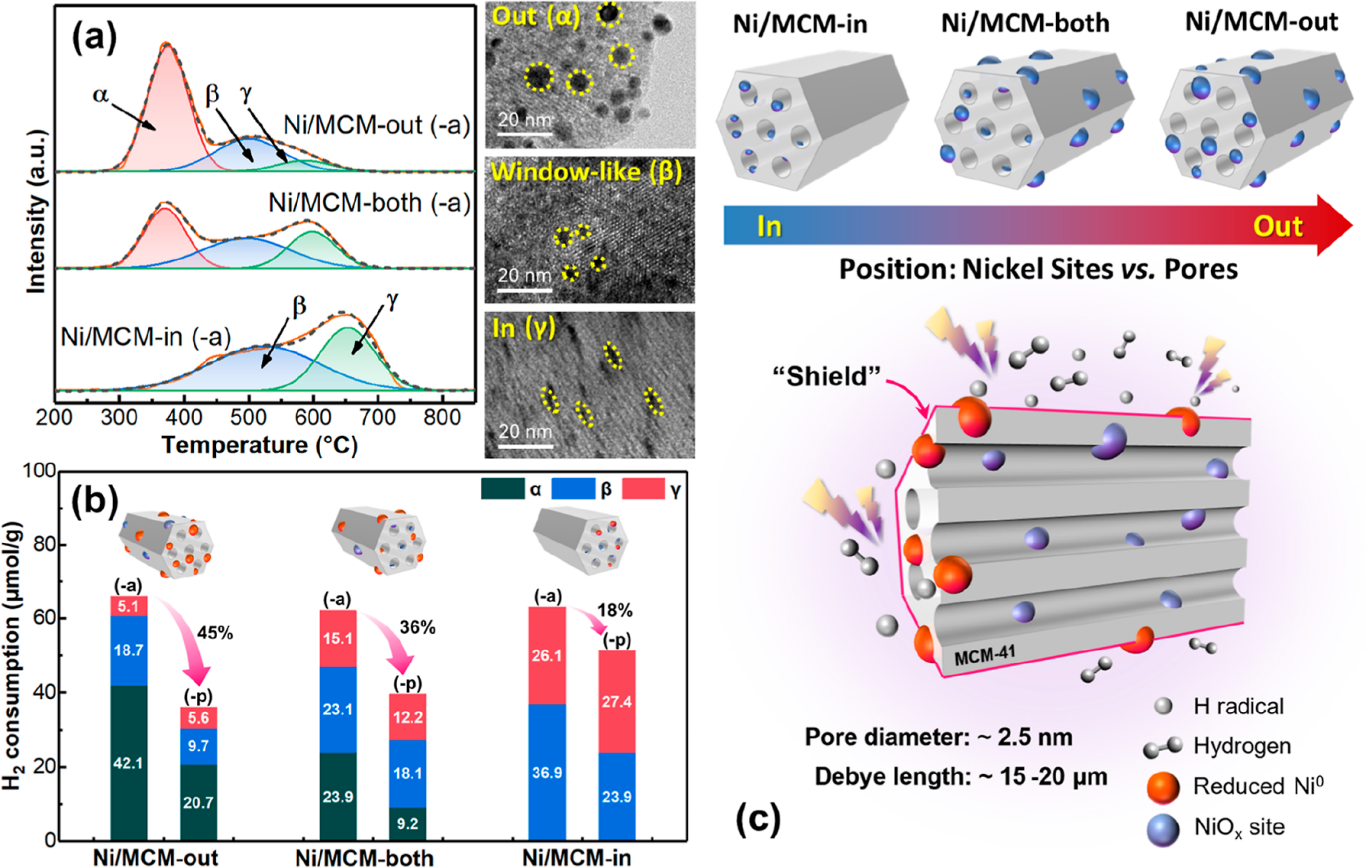
(a) H_2_-TPR of the as-prepared Ni/MCM-out, Ni/MCM-both, and Ni/MCM-in, and the corresponding TEM images for out (α), window-like (β), and in (γ) Ni sites; (b) H_2_ consumption (by TPR) for the as-prepared catalysts (-a) and the H_2_ plasma-treated catalysts (-p) (at a specific energy input, SEI, of 24 kJ/L, SEI = plasma discharge power over gas flow rate); and (c) schematics of the supported Ni catalysts on MCM-41 (top) and shielding effect of mesoporous MCM-41 on Ni species within its mesopores in an H_2_ plasma discharge.

The location of Ni sites in the catalysts was further evidenced by H_2_-temperature-programmed reduction (TPR) analyses. H_2_-TPR profiles of Ni/MCM-41 (Figure 2a) show three reduction peaks centered at about 350–400 °C (α), 500 (β), and 650 °C (γ), respectively, which correspond to the reduction of NiO species (i) on the external surfaces of the support (with a weak interaction with MCM-41), (ii) confined at the pore window (or the interlayer of catalyst particles), and (iii) in the mesopores of MCM-41, respectively.^26^ Specifically, Ni/MCM-out has the highest fraction for the α peak (∼65%), with the fraction of the γ peak being only ∼8% (Figures 2a and S3), implying that most NiO species are dispersed on the external surface of MCM-41. This can be proven by the observation of the Ni supported on the less porous SiO_2_ (i.e., the control catalyst, Ni/SiO_2_, Figures S4 and S5, Table S2), where the NiO species in the resulting catalyst are mostly dispersed on the external surface of SiO_2_ (about 80%, as shown in Figures S3 and S5). Comparatively, Figures 2a and S3 show that the NiO species in Ni/MCM-both are mainly confined to the pore windows (∼39%) and in the mesopores of MCM-41 (∼25%), whereas no NiO species are found on the external surface of Ni/MCM-in since the α peak was not detected.

Varying the Ni deposition locations on the catalysts affects the interaction between Ni sites and plasma discharge, which was proven through the study of the H_2_ plasma reduction of the catalysts (at 36 kJ/L for 3 h) and the following H_2_-TPR analysis (of the catalysts before and after H_2_ plasma treatment, Figures 2b and S6, Table S3). Compared to the corresponding as-prepared catalysts (Figure 2b), the H_2_ plasma-treated catalysts showed a lower total amount of H_2_ consumption (Figure 2b), decreasing in the following order: Ni/MCM-out (∼45%) > Ni/MCM-both (∼36%) > Ni/MCM-in (∼18%). This finding shows that the NiO species in the three catalysts could be reduced by the H_2_ plasma to different extents. In detail, about 51 and 62% of the NiO species on the external surface (α peak) of Ni/MCM-out and Ni/MCM-both were reduced, respectively, after the H_2_ plasma treatment, suggesting that the external NiO species of these catalysts are more easily reduced by the H_2_ plasma when compared to the NiO species at the pore window (β peak) and inside the mesopores (γ peak). Additionally, the H_2_ consumption due to the reduction of internal NiO species (γ peak) for all the catalysts was nearly unchanged after the plasma treatment, suggesting that the NiO species confined in the mesopores were hardly reduced at all by the reducing plasma discharge. These findings show that (i) Ni NPs distributed on the external surface of MCM-41 interact with the plasma more effectively when compared to Ni NPs confined in the mesopores and (ii) mesopores of MCM-41 can act as a shield due to the limited interaction between plasma discharge and species in the mesopores, as illustrated by schematics in Figure 2c.

Electrical diagnostics show that packing of the catalysts in the discharge enhanced the relevant discharge properties (Table 1, Figure S7 and Table S4). The Debye length (λ_D_) (calculation of λ_D_ is presented in the Supporting Information based on eq S8) of the plasma + packing systems increased to ∼20 μm compared to ∼16.5 μm in the plasma alone. The λ_D_ values of the discharge are nevertheless several orders of magnitude larger than the pores size of MCM-41 (∼2.5 nm), and thus, plasma discharge was unlikely to form in the mesopores of MCM-41, which is consistent with previous findings.^28,29^ In addition, plasma-induced reactive species rarely diffuse into the mesopores of MCM-41 due to their short lifetimes (on the scale of nanoseconds^30^), which makes the interaction between the plasma generated species and the confined NiO species impossible. These results explain the H_2_-TPR data (Figure 2b) discussed above, that is, the lower reduction degree of NiO species in the mesopores of MCM-41 in comparison to the NiO species on the external surface.^28,29^ As a result, it confirms that species in the mesopores of MCM-41 are shielded and protected during plasma discharge; this phenomenon can thus be used to design bespoke catalysts to improve plasma-catalytic NH_3_ synthesis. Specifically, we hypothesized that, by employing Ni/MCM-out in the hybrid plasma-catalytic system, Ni NPs on the external surface of mesoporous MCM-41 could enhance the surface reactions under plasma conditions, and the formed NH_3_ could then diffuse into mesopores of MCM-41 (due to the NH_3_ gradient across the framework in the reaction), where plasma is absent, avoiding dissociation via the plasma-induced reverse reaction.

**Table 1:** Discharge Properties of the Plasma alone and Plasma + Packing Systems^a^

packing	*E*^c^ (kV/cm)	*E*/*N*^c^ (Td)	*E*_e_^c^ (eV)	λ_D_^c^ (μm)
no packing^b^	28.3 ± 0.8	118.8 ± 3	4.8 ± 0.2	16.5 ± 0.2
MCM-41	37.9 ± 0.2	159.0 ± 8	5.9 ± 0.3	19.7 ± 0.7
Ni/MCM-in	38.5 ± 0.2	161.5 ± 7	6.0 ± 0.2	20.1 ± 0.3
Ni/MCM-out	39.6 ± 0.2	166.3 ± 7	6.1 ± 0.2	20.5 ± 0.4

a All experiments were performed at SEI = 24 kJ/L.

b The plasma alone.

c Calculations of the average electric field (*E*), reduced electric field (*E*/*N*), mean electron energy (*E*_e_), and Debye length (λ_D_) are described in the Supporting Information.

### Catalytic Performance

**Figure 3:**
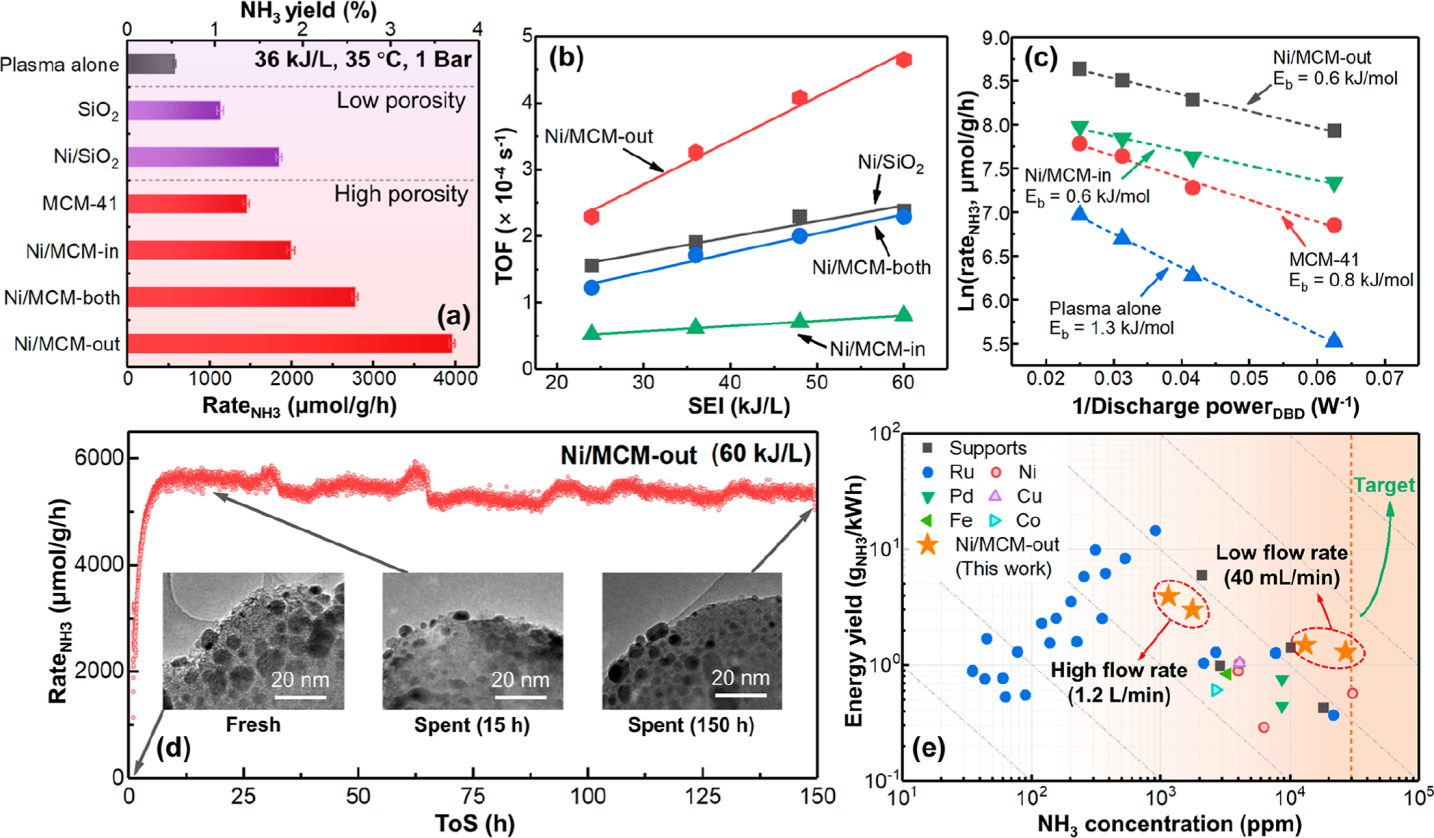
(a) *R*_NH_3__ and NH_3_ yield of plasma alone and plasma-catalytic systems (with SiO_2_, MCM-41 and catalysts based on them, SEI = 36 kJ/L, *Q*_gas_ = 40 mL/min, at 35 °C and 1 bar. Each experiment lasted 3 h). (Errors were derived from three tests under the same condition). (b) Turnover frequency (TOF, of plasma-catalytic systems) as a function of SEI. (c) Logarithmic reaction rate (ln(*R*_NH_3__)) vs 1/discharge power_DBD_ plots for the plasma alone and hybrid systems (based on the MCM-41 support and Ni/MCM-41 catalysts). (d) *R*_NH_3__ over Ni/MCM-out as a function of ToS for 150 h (The continuous sampling interval is 20 s). (e) Reported energy yield vs NH_3_ concentration in the plasma-catalytic NH_3_ synthesis over different metallic catalysts using DBD. Plotted experimental data are reproduced from the works of Bai et al.,^39^ Barboun et al.,^40^ Gómez-Ramírez et al.,^41^ Herrera et al.,^42^ Iwamoto et al.,^43,44^ Kim et al.,^45^ Li et al.,^46^ Mizushima et al.,^47,48^ Patil et al.,^49^ Peng et al.,^24,50,51^ Shah et al.,^23,36^ Wang et al.,^17^ Xie et al.,^52^ and Zhu et al.^53^

Compared to the plasma alone, packing the DBD reactor with SiO_2_ or MCM-41 supports enhanced the ammonia synthesis rate (*R*_NH_3__, as defined by eq S3), that is, 575 μmol/g/h (plasma alone) versus 1131 μmol/g/h (with SiO_2_) and 1452 μmol/g/h (with MCM-41), as shown in Figure 3a. The comparatively high *R*_NH_3__ value of the plasma-MCM-41 system could be ascribed to the highly porous structure of MCM-41 compared to SiO_2_ (Table S2).^31^ As expected, loading Ni onto either SiO_2_ or MCM-41 enhanced the *R*_NH_3__ significantly, proving the active role of metallic Ni in ammonia synthesis.^32^ Again, the Ni/MCM-41 catalysts were more active than Ni/SiO_2_ with the following activity order: Ni/MCM-out (3959 μmol/g/h) > Ni/MCM-both (2775 μmol/g/h) > Ni/MCM-in (1992 μmol/g/h) > Ni/SiO_2_ (1847 μmol/g/h). In particular, Ni/MCM-out exhibited a high NH_3_ yield of about 3.7%, which was ∼8 times higher than that of the plasma alone. Figure 3b shows that the turnover frequencies (TOFs) of all the supported Ni catalysts increased with an increase in SEI, while the TOF of these catalysts decreased in the following order: Ni/MCM-out > Ni/SiO_2_ > Ni/MCM-both > Ni/MCM-in at a constant SEI. Notably, for the Ni/MCM catalysts, the TOF values increased with an increase in the fraction of external Ni sites (Figure S9). Compared to Ni/MCM-in, the improved performance of Ni/MCM-out, Ni/SiO_2_, and Ni/MCM-both confirms that the enhanced interaction between externally accessible Ni NPs and plasma (as discussed above) is beneficial to plasma-catalytic NH_3_ synthesis. Again, in comparison with the control of Ni/SiO_2_ (TOF = 2.4 × 10^–4^ s^–1^ at 60 kJ/L), the higher TOF of Ni/MCM-out (i.e., 4.7 × 10^–4^ s^–1^ at 60 kJ/L) could be attributed to the highly porous structure of MCM-41 (Table S2).

Figure 3c shows a modified Arrhenius plot (eq 1) correlating ln(*R*_NH_3__) with the reciprocal of discharge power (1/*P*_d_) at 35 °C,^33^ which was used to determine the apparent activation energy (*E*_a_) in the plasma-catalytic ammonia synthesis. Compared to the plasma alone (*E*_a_ = 1.3 kJ/mol), the plasma with MCM-41 and Ni/MCM packing decreased the activation energy of the overall reaction to ∼0.8 and ∼0.6 kJ/mol, respectively. These findings suggest that the supported Ni sites of the Ni/MCM catalysts reduced the reaction barrier effectively, according to the Brønsted–Evans–Polanyi relationship, most likely due to the appropriate nitrogen binding energy on Ni surfaces.^20^ It is worth noting that the estimated values of *E*_a_ for Ni/MCM-in and Ni/MCM-out are almost the same, indicating that the dependence of the chemical rate coefficients on the discharge power is similar for both Ni-based catalysts.
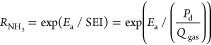
1where *P*_d_ is the discharge power and *Q*_gas_ is the total flow rate.

A longevity test lasting 150 h was performed to investigate the stability of the Ni/MCM-out catalyst. As shown in Figure 3d, Ni/MCM-out was very stable under plasma conditions, with final and initial *R*_NH_3__ values (i.e., 5380 ± 121 μmol/g/h in the final 150 h vs 5630 ± 118 μmol/g/h in the first 20 h) being quite comparable. A comparison of the small-angle X-ray diffraction (XRD) patterns (Figure S10a) and textural properties (Figure S10c,d and Table S5) of the fresh and spent Ni/MCM-out catalysts proves the intactness of highly ordered hexagonal mesopores in the catalyst after the 150 h reaction under plasma discharge.^34^ In addition, as shown in Figures 3d, S10b and S11, comparative high-angle XRD and HRTEM analyses of the fresh and spent Ni/MCM-out catalysts show that the Ni NPs on the external surface of MCM-41 remained stable without significant migration after 15 and 150 h of plasma reaction. Furthermore, the NH_3_ synthesis rate (*R*_NH_3__) of Ni/MCM-out remained constant over five reuse cycles (Figure S12a), and Ni/MCM-out also exhibited excellent stability over 30 h of time-on-stream (ToS), even under the conditions of switching SEIs (between 36 and 60 kJ/L) during operation (Figure S12b).

Figure S13 shows that the energy yield of ammonia synthesis over the Ni/MCM-41 catalysts decreased with an increase in SEI from 24 to 60 kJ/L. The highest energy yield of 1.2 g_NH_3__/kWh was achieved over Ni/MCM-out, ∼12 times that using plasma alone and ∼3 times that over MCM-41 at an SEI of 24 kJ/L. Figure S14 presents the influence of SEI (at a fixed discharge power of 16 or 32 W and different total flow rates) on the performances of the hybrid system, demonstrating that increasing the flow rate (or decreasing the SEI) resulted in a decrease in NH_3_ concentration (e.g., from 23684 to 1772 ppm at 32 W, Figure S14a) and an increase in energy yield (Figure S14b).

NH_3_ concentration is a key factor for downstream NH_3_ separation, which is known to be one of the most energy-intensive processes in ammonia synthesis.^35^Table S6 presents a comprehensive comparison of the performance of the state-of-the-art plasma-assisted NH_3_ synthesis systems, demonstrating that the system employing the Ni/MCM-out catalyst represents one of the best at achieving high NH_3_ concentrations.

A comparison of the ammonia concentration and energy yield for various metallic catalysts is given in Figure 3e. Ruthenium (Ru), as the most studied metal in plasma-catalytic ammonia synthesis, shows a relatively higher energy yield but with a very low ammonia concentration (<1000 ppm), whereas other metals, such as Ni and Pd, could achieve a higher ammonia concentration with a lower energy yield (<1 g_NH_3__/kWh). As noble catalysts are relatively expensive, the plasma-catalytic system using Ni/MCM-out displays very promising performance (at a low flow rate of 40 mL/min) with a high NH_3_ yield (5.3%) and concentration (27115 ppm) at an energy yield of 1.2 g_NH_3__/kWh. The energy yield of the Ni/MCM-out system could be improved to 3.9 g_NH_3__/kWh by increasing the flow rate to 1.2 L/min, but this operation compromises the ammonia concentration. A comparison with the relevant work using porous materials (including MOFs and zeolites)^23,24,36−38^ was also performed, as shown in Figure S15a,b, in which the developed Ni/MCM-out system exhibits the highest NH_3_ concentration alongside a competitive energy yield.

### Surface NH_*x*_ Species Formation on External Ni Sites

**Figure 4:**
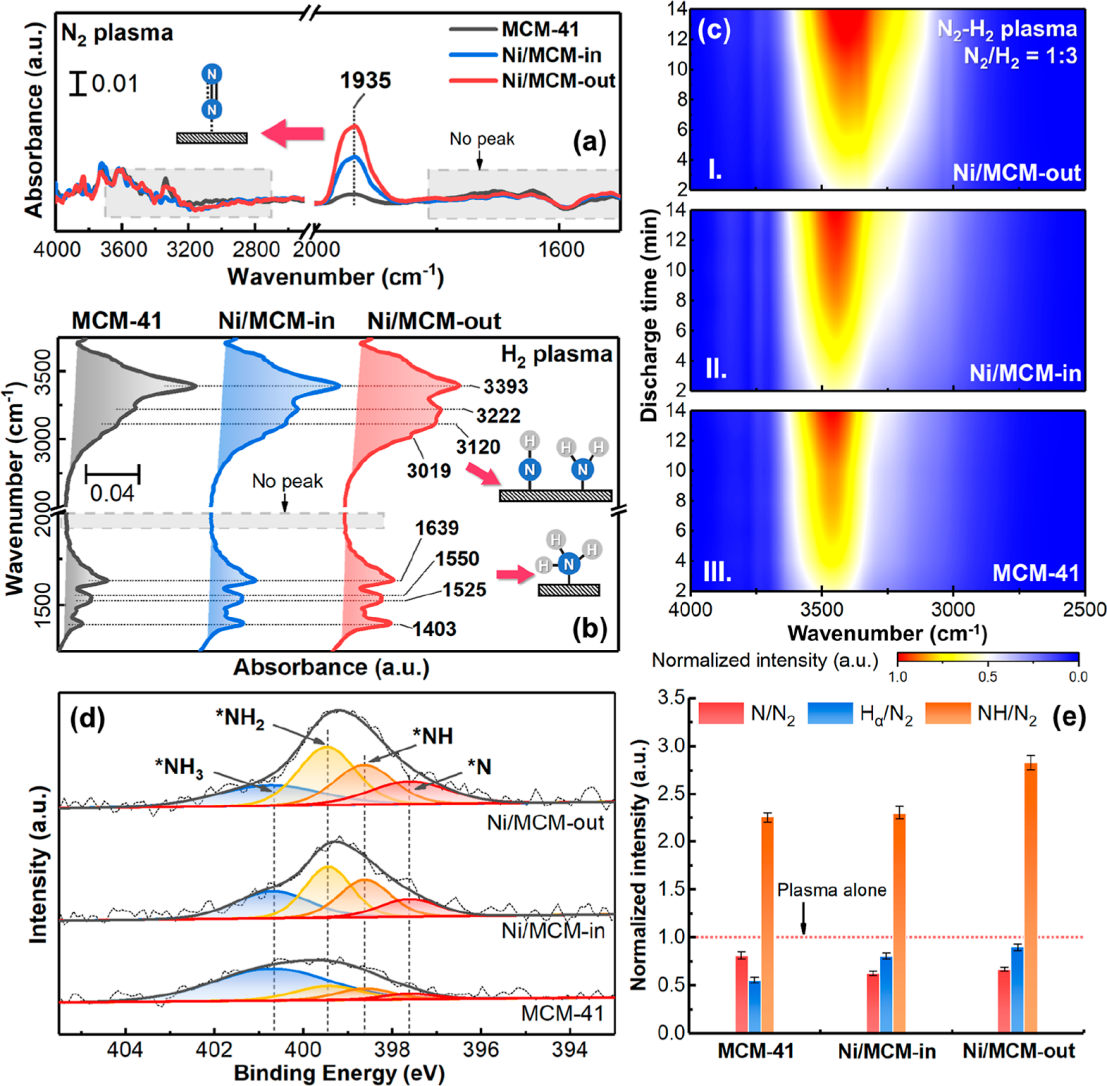
In situ FTIR spectra of (a) N_2_ activation and adsorption (in N_2_ plasma, *Q*_gas_ = 40 mL/min) and (b) hydrogenation of the adsorbed N_2_ (in an H_2_ plasma, *Q*_gas_ = 40 mL/min) at 5 min of discharge time. (c) IR spectra of plasma-assisted ammonia synthesis (discharge time = 15 min, *Q*_gas_ = 40 mL/min, N_2_/H_2_ = 1:3, full scale as shown in Figure S17) on MCM-41, Ni/MCM-in, and Ni/MCM-out, respectively. (d) N 1s XPS profiles of the spent catalysts and MCM-41 after the reaction. (e) Normalized relative intensities of N, H_α_, and NH peaks as measured by optical emission spectrometry (SEI = 24 kJ/L, 35 °C, 1 bar, full scale as shown in Figure S20a) (Errors were obtained from three tests under the same conditions).

We have shown that the Ni/MCM-out catalyst performed best in the plasma-catalytic synthesis of NH_3_. Accordingly, in situ Fourier-transform infrared spectroscopy (FTIR) characterization (Figure S16, relevant experimental details are in Supporting Information) was carried out to gain insight into the plasma-assisted surface reactions and prove the proposed hypothesis. Regarding surface intermediates on MCM-41 and Ni/MCM-in/-out in N_2_ (corresponding IR spectra and information are shown in Figure S17 and Table S7), Figure 4a shows a distinctive band at 1935 cm^–1^, which is attributed to N_2_ stretching of the end-on adsorbed *N_2_ molecules on the Ni surface in the plasma,^54^ suggesting that the plasma could enable N_2_ activation and adsorption on Ni even at room temperature. Among these three catalysts, the N=N stretching band of Ni/MCM-out shows the highest relative intensity, proving that the external Ni sites on Ni/MCM-out are readily available and accessible for interactions with N_2_ and plasma discharge, which significantly enhances the formation of adsorbed *N_2_.

Hydrogenation of the adsorbed N_2_ was investigated using in situ FTIR. In detail, before plasma ignition, the catalyst was exposed to pure N_2_ for 10 min, followed by an H_2_ flush to create the Ni surfaces with a small amount of N_2_ molecules adsorbed at room temperature. Figure 4b shows that H_2_ plasma was able to break the N≡N triple bond of the adsorbed N_2_ on the Ni surface, forming coordinated *NH_3_ (ν_as_(NH_3_): 3393 cm^–1^; ν_s_(NH_3_): 3222 cm^–1^; δ_as_(NH_3_): 1639 cm^–1^; δ_as_(NH_3_) or δ_as_(NH_4_^+^): 1550, 1525 cm^–1^). A small IR peak at 1403 cm^–1^ represents *NH species, which is one of the critical intermediates in the surface reactions of ammonia synthesis.^55^ Compared to the bare MCM-41, the metallic Ni sites, especially on the external surface of MCM-41, promoted the formation of surface *NH_*x*_ (*x* = 1 and 2) amide groups significantly, as indicated by the increased intensity of the characteristic IR peaks at 3120 cm^–1^ (for ν_as_(NH_*x*_)), 3019 cm^–1^ (for ν_s_(NH_*x*_)), and 1403 cm^–1^ (Ni–*NH_*x*_). Note that the IR peaks of N=N stretching between 1900 and 2000 cm^–1^ are absent, which reveals that in the presence of H and H_2_ radicals, *N_2_ species could be rapidly dissociated and hydrogenated to form *NH_*x*_ species on the catalyst surface, instead of forming adsorbed *N_2_H_*x*_ species (which are the major intermediates in the electrochemical nitrogen reduction reactions^56,57^). Furthermore, the transformation of the surface-adsorbed species in the N_2_–H_2_ plasma (N_2_/H_2_ = 1:3) was also monitored using in situ FTIR. The IR spectra of these three catalysts show similar assignable peaks of coordinated NH_3_ on the catalyst surfaces. In the system over Ni/MCM-out, the intensity of the peaks at 3000–3250 and 1400–1470 cm^–1^ (Figures 4c and S17) was the strongest among the systems under investigation. This result suggests that the external Ni sites of Ni/MCM-out promoted the formation of *NH_*x*_ species, particularly the *NH group (1414 cm^–1^, Figure S18), in agreement with the aforementioned hypothesis, that is, externally accessible Ni sites are critical to enabling the effective interaction with plasma and thus the catalytic surface reactions.

Additionally, N 1s X-ray photoelectron spectroscopy (XPS) profiles of the spent catalysts and MCM-41 after the plasma reaction (Figure 4d and Table S8) also revealed the presence of adsorbed *NH_*x*_ (where *x* = 0, 1, 2, or 3) species on their surfaces. The predominant N-containing adsorbed species on the bare MCM-41 are *NH_3_ with a fraction of ∼67%, whereas the surface proportions of *NH_3_ on Ni/MCM-in and Ni/MCM-out are 28 and 23%, respectively. For Ni/MCM-in and Ni/MCM-out, all *NH_*x*_ (*x* = 0, 1, 2, or 3) species can be detected after the reaction, and the fractions of *NH and *NH_2_ are higher than those on the surface of MCM-41, which supports the analysis of the in situ IR spectra (Figure 4b,c). Importantly, Ni/MCM-out showed a higher fraction of *N (∼19%) compared to Ni/MCM-in, demonstrating that the external Ni sites were highly beneficial to the dissociation of adsorbed *N_2_ molecules under plasma conditions (Figure 4a).

Corresponding to the surface species via the IR spectra, the emission spectra of the N_2_–H_2_ DBD show numerous excited N_2_ molecular bands, including N_2_ (C^3^Π_u_ → B^3^Π_g_ and B^3^Π_g_ → A^3^Σ_u_) and N_2_^+^ (B^2^Σ_u_^+^ → X^2^Π_g_^+^), suggesting that electronic excitation and ionization of N_2_ took place in the N_2_–H_2_ plasma (Figures S19 and S20a). In addition, the presence of N (3p^2^P_0_–3s^2^P) atomic lines and H_α_ Balmer lines confirms the dissociation of N_2_ and H_2_, which could be driven by the initial electron impact reactions of N_2_ and H_2_ in the discharge (Figure S20b). The DBD with Ni/MCM-in has a similar mean electron energy (∼6.0 eV) as the discharge coupled with Ni/MCM-out, resulting in a similar distribution of four energy loss channels of N_2_ and H_2_. Among these four energy loss channels, electronic excitations over Ni/MCM-out are the most important for both N_2_ and H_2_, accounting for 55 and 77%, respectively (Figure S8). Furthermore, vibrational excitation and dissociation are also essential for N_2_ molecules. The relative intensities of N and H_α_ atomic lines, as well as the gas-phase NH band, are normalized to the intensities of the plasma alone. As shown in Figure 4e, the presence of the catalysts increased the relative intensity of NH but decreased the intensity of both N and H lines compared to the plasma alone, indicating that the catalysts promoted the formation of intermediates (e.g., NH) and the adsorption of N and H species onto the surface. Importantly, when compared to bare MCM-41, packing Ni/MCM-out in the DBD generated more fractions of H and NH radicals with fewer N radicals in the gas phase, indicating that N_2_ molecules could interact with the Ni surface relatively easily when the Ni surface is more accessible, that is, dispersed on the external surface of MCM-41. These findings also suggest that the conversion of adsorbed *N_2_ species to *NH_*x*_ (*x* = 1 or 2) could be much easier on the Ni surface than in the gas phase.

### Proposed Mechanism

**Figure 5:**
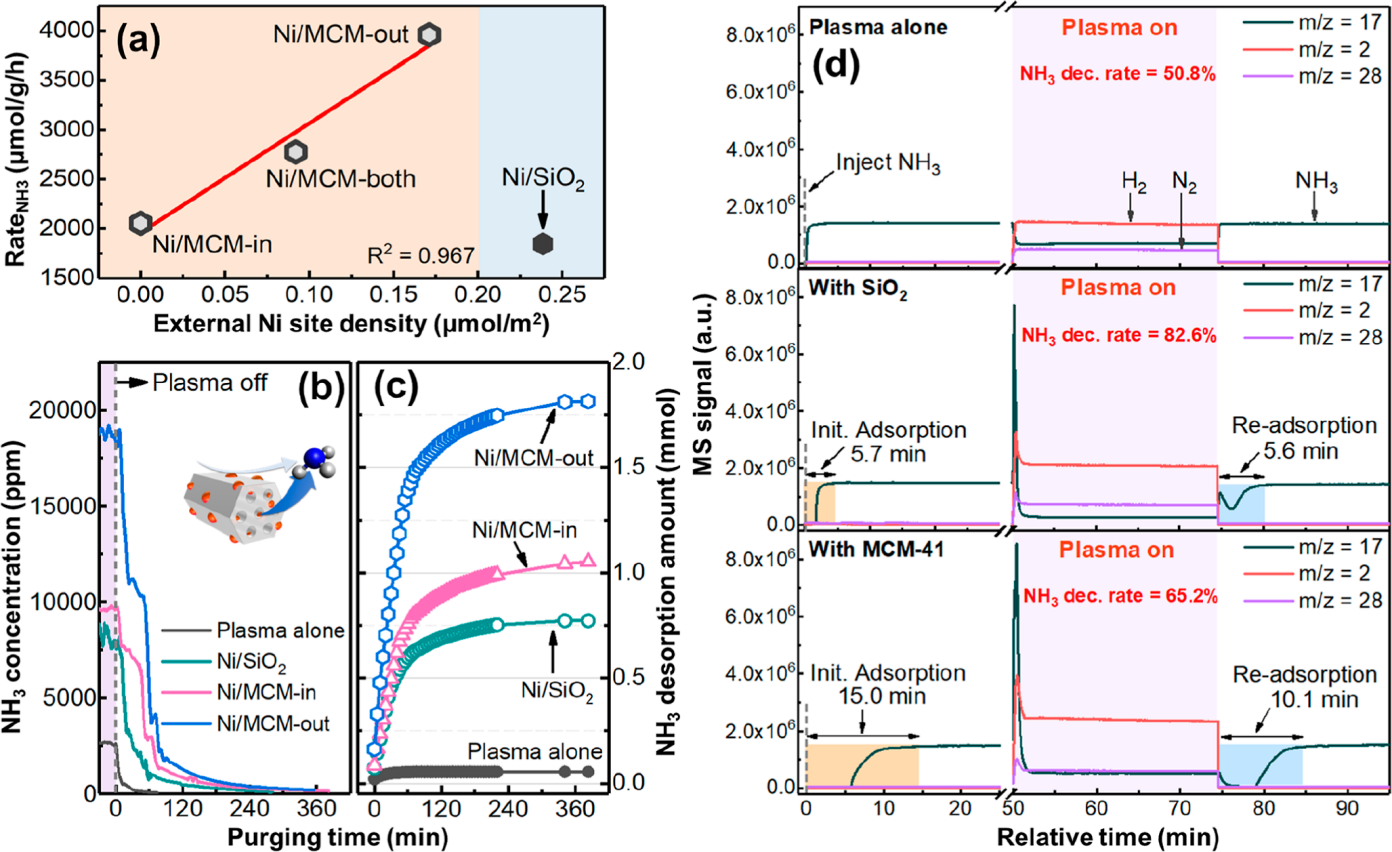
(a) NH_3_ synthesis rate vs the external Ni site densities of different supported Ni catalysts on SiO_2_ and MCM-41 (at 36 kJ/L, 35 °C and 1 bar). (b) Desorbed NH_3_ concentration and (c) corresponding accumulated amount vs purging time in the plasma alone and plasma-catalyst systems (catalyst amount: ∼0.5 g, mixed N_2_/H_2_ as the purge gas). (d) Plasma-driven decomposition of NH_3_ in the plasma alone and plasma-SiO_2_/-MCM-41 systems (init. adsorption: initial adsorption stage; re-adsorption: NH_3_ re-adsorption stage; NH_3_ dec. rate: NH_3_ decomposition rate; discharge power = 5 W, with ∼0.1 g catalysts).

Figure 5a shows a clear linear correlation between *R*_NH_3__ and the density of external Ni sites of the Ni/MCM-41 catalysts (the density is defined by eq S10 and shown in Table S9). Ni/MCM-out with the highest external Ni site density of 0.17 μmol/m^2^ exhibited the highest *R*_NH_3__ value of 3959 μmol/g/h. Interestingly, the control catalyst of Ni/SiO_2_ possessed a very high external Ni density at 0.24 μmol/m^2^ but a much lower ammonia synthesis rate of 1511 μmol/g/h. These findings suggest that the combination of the mesoporous structure of MCM-41 and the externally supported Ni sites is critical for boosting the efficiency of the plasma-catalytic NH_3_ synthesis.

Ordered mesoporous silicas, such as MCM-41, are effective adsorbents for ammonia due to their high adsorptive potential.^58^ Our findings show that the Ni/MCM catalysts could adsorb the generated ammonia in situ during the plasma catalysis. This was evidenced by ammonia desorption from the catalysts when the plasma was turned off (using mixed N_2_/H_2_ as the purge), as shown in Figure 5b,c. In the hybrid system with a catalyst, the initial desorption of ammonia was rapid (in the first hour), and then, it levelled off. Complete desorption of ammonia from Ni/MCM-in and Ni/MCM-out took more than 6 h, whereas the desorption ended at only 30 min for the plasma alone. Figure 5c shows that the total amount of desorbed ammonia from the system using Ni/MCM-out was the highest at ∼0.91 mmol/g, suggesting that the design strategy of supporting active Ni on the external surface of mesoporous MCM-41 significantly favored the preservation of the formed NH_3_ under plasma conditions. Specifically, Ni/MCM-out promoted the high formation of NH_3_ (due to the externally supported Ni), which could create a high local NH_3_ concentration gradient across the MCM-41 framework in a dynamic equilibrium state, being favorable in promotion of the diffusion of the formed NH_3_ into the internal mesoporous space of MCM-41. Hence, the intake of NH_3_ into the internal mesoporous space of MCM-41, where plasma discharge is absent during the plasma-catalytic reaction, protected the produced ammonia from the plasma-induced reverse reaction (NH_3_ decomposition), enabling the hybrid system employing Ni/MCM-out to achieve such a high performance. Accordingly, here we interpreted this unique phenomenon as the “shielding protection” of the mesoporous MCM-41 framework for preventing the decomposition of the formed NH_3_.

To prove this hypothesis further, we investigated plasma-induced NH_3_ decomposition in different DBD systems (with a continuous feed of 2.5 vol % NH_3_ in Ar balance), that is, plasma alone, plasma + SiO_2_, and plasma + MCM-41. As shown in Figure 5d, during the initial adsorption stage, the systems with SiO_2_ and MCM-41 packing required about 5.7 and 15.0 min, respectively, to reach saturation. At 50 min, when plasma discharge was initiated, sharp and intense NH_3_ peaks were measured for the systems with SiO_2_ and MCM-41, which proved that the adsorbed NH_3_ (in the bare supports) was rapidly desorbed due to the discharge. When the plasma was turned off, the systems using SiO_2_ and MCM-41 required more time to resaturate than the plasma alone. Notably, for the plasma system packed with SiO_2_, the time required for resaturation was almost the same as that required for the initial saturation, indicating that the plasma discharge removed the majority of the adsorbed NH_3_. In contrast, the resaturation time required by MCM-41 was reduced by ∼33% compared to its initial saturation stage, demonstrating that MCM-41 was able to retain a portion of the adsorbed ammonia during plasma discharge. The findings show that the internal space of mesopores of MCM-41 indeed served as a shelter for NH_3_ from being decomposed by plasma discharge, that is, the “shielding protection” provided by mesoporous MCM-41. In addition, when the system was in equilibrium under plasma discharge, the decomposition of NH_3_ (*m*/*z* = 17) in the system using MCM-41 was ∼65.2%, lower than that using SiO_2_ (∼82.6%), which indicates that part of the ammonia molecules did enter the mesopores of MCM-41 and were thus protected from the plasma-induced reverse reaction (NH_3_ decomposition).

**Figure 6:**
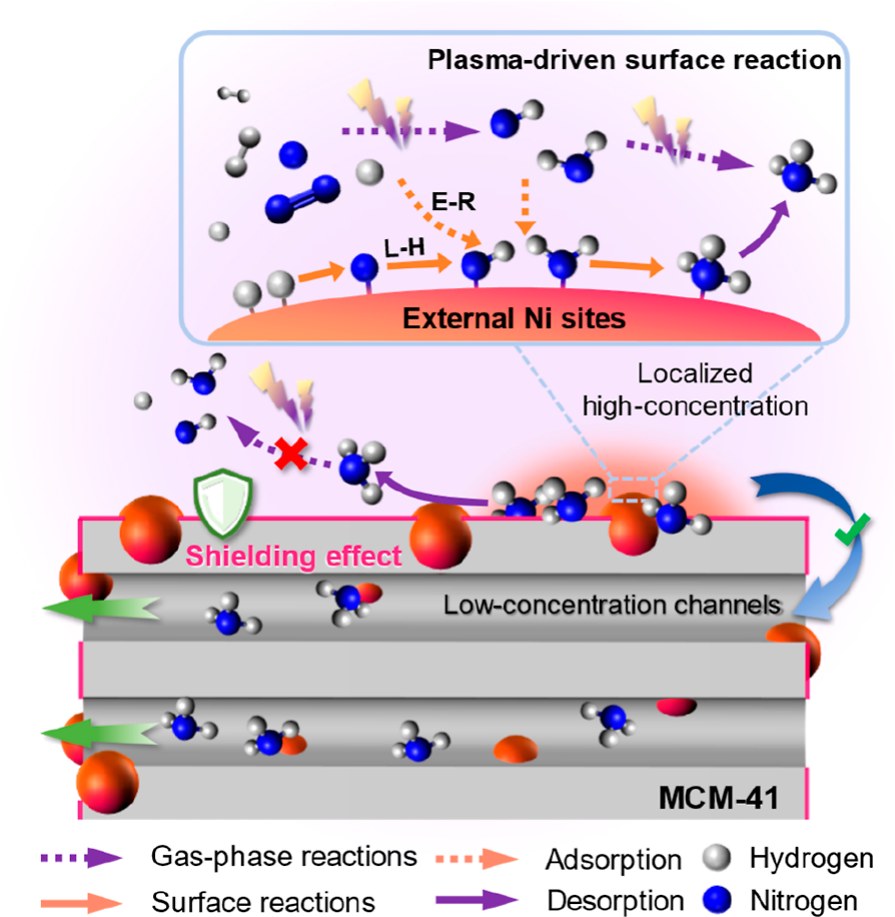
Schematic of the proposed mechanism for the plasma-assisted surface reaction and the “shielding protection” effect of mesoporous MCM-41.

The supported Ni catalysts on mesoporous MCM-41 have shown excellent activity in the plasma-catalytic ammonia synthesis, especially Ni/MCM-out with the Ni NPs mainly dispersed on its external surface, which is readily accessible for the plasma discharge, enabling the effective interaction between plasma and surface-adsorbed species. Importantly, the formed ammonia could diffuse into the internal mesopores of MCM-41, which could be due to the concentration gradient across its framework and thus avoid the plasma-induced decomposition, that is, the “shielding” effect of the mesoporous structure of MCM-41. Accordingly, a mechanism can be deduced from the findings above, as illustrated in Figure 6.

The mechanism was supported by the comprehensive characterization of the hybrid plasma-catalytic system under investigation. XPS analysis of the catalysts and in situ FTIR characterization (Figure 4a–d) showed that the external Ni sites enhanced the adsorption and dissociation of N_2_ to form adsorbed *N_2_ and *N species initially, which facilitated the formation of NH_*x*_ (*x* = 1 and 2) intermediates via hydrogenation with either *H or H radicals.^17^ Furthermore, a significant amount of *NH_3_ was retained on the surfaces of the spent Ni/MCM-41 catalysts (Figure 4d), which proved that the abundant internal surfaces of mesoporous MCM-41 could effectively store, and more importantly, protect the generated NH_3_ from being decomposed by the plasma-induced reverse reaction during the plasma-catalytic process. The desorbed NH_3_ off the external Ni sites could induce a localized high concentration of ammonia over the external surface of the Ni/MCM catalysts. Consequently, the concentration gradient of ammonia across the mesoporous framework of MCM-41 (note that the internal NH_3_ concentration in MCM-41 is relatively low due to the absence of the plasma in the mesopores) may be common, enhancing ammonia diffusion from the external Ni sites into the mesopores of MCM-41 (Figure 6). According to Le Chatelier’s principle, the diffusion of NH_3_ from the external Ni sites into the mesopores could prevent the plasma-induced reverse reaction (i.e., NH_3_ decomposition) and shift the reaction equilibrium toward the production of ammonia. In conclusion, the interaction of Ni sites and plasma is enhanced due to the unique design of the Ni catalyst supported on the external surface of mesoporous MCM-41. More importantly, plasma cannot form in or penetrate into the mesopores during the plasma-catalytic reaction; hence, the mesoporous structure of the Ni/MCM catalysts provides a unique “shielding” effect, which can effectively reduce the reverse reaction (NH_3_ decomposition) in the hybrid system, contributing significantly to the enhanced ammonia synthesis rate.

## Conclusions

We demonstrated a promising catalyst design strategy based on the “shielding protection” mechanism using the mesoporous structure of Ni/MCM-41 that enables the enhanced plasma-catalytic synthesis of ammonia at ambient conditions. The catalyst with Ni NPs on the external surface of MCM-41 (Ni/MCM-out) showed an impressive ammonia yield of >5% and a balanced energy yield of 1.5 g_NH_3__/kWh. The findings of this study reveal that metallic Ni sites with good accessibility (i.e., on the external surface of MCM-41) are critical for enhancing the activation of N_2_ molecules and promoting the formation of adsorbed *NH_*x*_ (*x* = 0, 1, and 2), thereby accelerating the intrinsic NH_3_ synthesis rate. Moreover, the internal space of the mesoporous MCM-41 support, which is exempt from plasma discharge, can provide “shielding protection” for the formed NH_3_, thus limiting the reverse reaction (i.e., ammonia decomposition) and shifting the reaction equilibrium to enhance NH_3_ production under plasma conditions. These two aspects were experimentally proven by comprehensive in situ FTIR and ex situ XPS characterization of the hybrid systems and catalysts. This work provides new insights into the mechanism of the plasma-catalytic NH_3_ synthesis over mesoporous catalysts and demonstrates the importance of rational design of tailored catalysts for plasma catalysis, which is critical to advancing the development of hybrid plasma-catalytic systems toward practical adoption.

## Experimental Section

### Catalyst Preparation

The supported Ni catalysts on MCM-41 were prepared using the synthesis methods described previously.^59,60^ Ni NPs (10 wt % loading) were deposited in different locations of MCM-41, including only in its mesopores, mainly on its outer surface, and across its framework, which are labeled as Ni/MCM-in, Ni/MCM-out, and Ni/MCM-both, respectively. Briefly, for the synthesis of Ni/MCM-in, the MCM-41 support was prepared first using the following procedure. Cetyltrimethylammonium chloride (CTAC, 25 wt %) was mixed with tetraethyl orthosilicate (TEOS, 99.99 wt %) and ammonium hydroxide solution (NH_3_·H_2_O, 33 wt %) with a volume ratio of 1:1:0.9 in 200 mL of deionized water and stirred at room temperature for 1 h until it reached a gel state. The white sediment was collected by filtration, washed, and then dried at 80 °C overnight; this was followed by calcination at 550 °C for 6 h using a heating rate of 1 °C/min to obtain MCM-41. MCM-41 was then added to a Ni(NO)_3_ precursor solution (10 wt % in ethanol) under vigorous stirring. After the slow evaporation of the mixture at 80 °C, the obtained solid sample was calcined using the same parameters to obtain Ni/MCM-in. Ni/MCM-out was synthesized by mixing CTAC, TEOS, and NH_3_H_2_O with Ni(NO)_3_ in 200 mL of deionized water simultaneously, followed by the same drying and calcination process. For Ni/MCM-both, the precursor solution with a mixture of CTAC, TEOS, NH_3_H_2_O, and Ni(NO)_3_ in 200 mL of deionized water was prepared first. After vacuum filtration and calcination, the obtained solid sample was added into a Ni(NO)_3_ precursor solution (5 wt % in ethanol) and then evaporated slowly at 80 °C before the calcination at 550 °C for 6 h to get the Ni/MCM-both catalyst.

To prepare the control catalyst based on SiO_2_, commercial amorphous SiO_2_ (Thermo Scientific) was used as the support, and Ni/SiO_2_ with 10 wt % loading was prepared by the incipient wetness impregnation using nitrate salts (Alfa Aesar, 99.5%) as the metal precursor. SiO_2_ powder (3 g) was added to the solution of nitrate salts. The slurry was continuously stirred at 60 °C for 2 h, after which it was aged overnight at room temperature. The sample was then dried at 110 °C for 5 h and calcined at 550 °C for 6 h.

All the calcined catalytic samples were then sieved to 60–70 meshes, denoted as the as-prepared catalysts. For plasma-catalytic ammonia synthesis, the as-prepared catalysts were reduced by Ar/H_2_ mixed gas (100 mL min^–1^; Ar/H_2_ = 1:1) at 750 °C for 2 h before the plasma reaction (denoted as the reduced catalysts). Unless otherwise specified (e.g., the as-prepared catalysts and H_2_ plasma-treated catalysts), all the supported Ni catalysts (Ni/SiO_2_, Ni/MCM-in, Ni/MCM-41-both, and Ni/MCM-out) mentioned in this paper refer to the reduced catalysts (fresh catalysts after reduction and before reaction).

### Plasma-Assisted NH_3_ Synthesis

The experiments were conducted in a coaxial DBD reactor with a special ground electrode, as described previously.^17^ Compared to traditional DBD reactors using metal as the ground electrode, the reactor used water as the ground electrode, as well as for the cooling of the reactor. Water was circulated between two concentric quartz tubes using a circulation bath (Grant LT Ecocool 150) to maintain a reaction temperature of 35 °C during the experiments. Unlike liquid plasma or plasma-in-liquid reactors, where the plasma interacts with liquid directly, in this reactor design, the circulating water did not come into contact with the plasma and reactants during the reaction. The discharge length was about 50 mm, and the discharge gap was 1 mm. The DBD reactor was connected to an AC high voltage power supply with a peak voltage of up to 25 kV. The discharge power was controlled between 16 and 40 W, and the frequency was fixed at 9.2 kHz. N_2_ and H_2_ (1:3) were used as the discharge gas at a total flow rate of 40 mL/min. About 500 mg of the reduced catalyst was used for each experiment. The applied voltage of the DBD was measured by a high-voltage probe (TESTEC, HVP-15HF), while the current was recorded by a current monitor (Bergoz, CT-E0.5). The voltage on the external capacitor was measured to determine the charge formed in the DBD. All the electrical signals were confirmed by a four-channel digital oscilloscope (Tektronix, MDO 3024). The discharge power was calculated by using a typical *Q*-*U* Lissajous method. A system made in-house was used to monitor and control the discharge power of the DBD reactor during experiments. The temperature in the discharge zone was measured using a fiber optical thermometer (Omega, FOB102). For the control experiments of plasma alone and plasma-SiO_2_/MCM-41, the same process parameters were used.

### In Situ FTIR Characterization

An integrated DBD/gas cell (Figure S16a) was designed in-house for in situ FTIR (transmission mode) probing of plasma-assisted surface reactions in ammonia synthesis. The experiment was performed using a Jasco FT/IR-4600 FTIR spectrometer equipped with a Peltier stabilized DLaTGS detector with a resolution of 0.7 cm^–1^ using 32 scans. The DBD unit consisted of two round metal plates (high voltage electrode and ground electrode, respectively) with a diameter of 24 mm and a hole (Φ = 5 mm) in the center of the plates (for IR incident radiation). A quartz dielectric layer with a diameter of 24 mm and a thickness of 3 mm was placed between the two metal plates. The DBD reactor was cooled by introducing circulating water to the ground electrode to maintain the reaction temperature at ∼35 °C (being identical to that in the plasma reaction), thus excluding the thermal effect of the plasma in ammonia synthesis. The power supply was the same as when testing catalytic activity, which can provide AC high voltage with a peak voltage of up to 25 kV. In the in situ tests, the SEI was maintained at 24 kJ/L (16 W, 40 mL/min), and the frequency was fixed at about 9.2 kHz.

The catalyst was first reduced by 50 vol % H_2_/Ar at 750 °C for 2 h and then pressed into the form of a thin wafer (Φ = 10 mm) with a thickness of ∼0.3 mm. The thin wafer was then placed between the high voltage electrode and the quartz plate in the DBD reactor (the yellow box in Figure S16a) with a discharge gap of 1 mm, and thus, the plasma can be formed (Figure S16b) on the catalyst wafer. The DBD reactor was fixed in the gas cell, which was capped at both ends by IR-transparent KBr windows. Meanwhile, for in situ FTIR analysis, three experimental procedures (A, B, and C) were designed to investigate three different processes in the plasma-catalytic ammonia synthesis, that is, A: adsorption of N_2_ on a catalyst (in N_2_ plasma), B: hydrogenation of the adsorbed N_2_ (in H_2_ plasma), and C: plasma-assisted ammonia synthesis (in N_2_–H_2_ plasma), respectively. Detailed experimental procedures are described in the Supporting Information, Section S9.

### Plasma-Induced NH_3_ Decomposition

Plasma-induced decomposition of NH_3_ from SiO_2_ and MCM-41 was carried out to illustrate the shielding effect of the mesoporous structure of MCM-41 on plasma-induced NH_3_ decomposition. In detail, ∼0.1 g SiO_2_ or MCM-41 pellets (40–60 mesh) were packed into the DBD reactor and pre-treated by Ar for 15 min at a discharge power of 5 W to clean the support surface. Then, 2.5 vol % NH_3_ (balanced with Ar and using Kr as the internal standard) was fed into the reactor at 60 mL/min continuously during each test. Ar was used to avoid the signal saturation of mass spectrometry (MS) measurements. After complete saturation of the samples, the plasma (at 5 W) was ignited (at 50 min) to initiate plasma decomposition of the adsorbed NH_3_ (for 25 min). After that, the plasma was switched off, and a continuous feed of mixed NH_3_/Ar/Kr flow enabled the re-adsorption of SiO_2_/MCM-41. Variation of NH_3_, N_2_, and H_2_ concentrations from the reactor during experiments was monitored by online MS (HIDEN HPR-20).

## Supporting Information Available

Catalyst characterization, electrical signal analysis and plasma diagnostics, calculation of electron density and Debye length, TOF vs external Ni sites, stability tests of Ni/MCM-out, energy efficiency of plasma-assisted synthesis of ammonia, high flow rate tests of Ni/MCM-out, literature comparison of plasma-assisted ammonia synthesis, in situ FTIR characterization of the catalyst surface under plasma discharge, supplementary results of OES diagnostics, and calculation of the density of external Ni sites (PDF)

## Dedication

Dedicated to Prof. Jianzhong Chen on the occasion of his 70th birthday.
