# Supplementary material for: Shielding Protection by Mesoporous Catalysts for Improving Plasma-Catalytic Ambient Ammonia Synthesis
Source: J Am Chem Soc. 2022 Jun 22;144(27):12020–31. doi: 10.1021/jacs.2c01950 (PMC9284550; doi:10.1021/jacs.2c01950)
Supplement: Supplementary file 1 — ja2c01950_si_001.pdf [file ja2c01950_si_001.pdf]

Shielding protection by mesoporous catalysts for improving plasma-catalytic ambient ammonia synthesis

Supplementary Information (SI)

Yaolin Wang[1], Wenjie Yang[2], Shanshan Xu[3], Shufang Zhao[2], Guoxing Chen[4], Anke Weidenkaff[4,5], Christopher Hardacre[3], Xiaolei Fan[3], Jun Huang[2]*, and Xin Tu[1]**

[1] Department of Electrical Engineering and Electronics, University of Liverpool, Liverpool L69 3GJ, United Kingdom

[2] School of Chemical and Biomolecular Engineering, Sydney Nano Institute, The University of Sydney, Sydney, NSW, 2037, Australia

[3] Department of Chemical Engineering, School of Engineering, The University of Manchester, Oxford Road, Manchester, M13 9PL, United Kingdom

[4] Fraunhofer Research Institution for Materials Recycling and Resource Strategies IWKS, Brentanostraße 2a, 63755 Alzenau, Germany

[5] Department of Materials and Earth Sciences, Materials and Resources, Technical University of Darmstadt, Alarich-Weiss-Str. 2, 64287 Darmstadt, Germany

*Corresponding authors: xiaolei.fan@manchester.ac.uk (X.F.); jun.huang@sydney.edu.au (J.H.); xin.tu@liverpool.ac.uk (X.T.)

Table of Contents:

1. Supplementary information of catalyst characterization

2. Electrical signal analysis and plasma diagnostics

3. Calculation of electron density and Debye length

4. TOF vs external Ni sites

5. Stability tests of Ni/MCM-out

6. Energy efficiency of plasma-assisted synthesis of ammonia

7. High flow rate tests of Ni/MCM-out

8. Literature comparison of plasma-assisted ammonia synthesis

9. In situ FTIR characterization of the catalyst surface under plasma discharge

10. Supplementary results of OES diagnostics

11. Calculation of the density of external Ni sites

1. Supplementary information of catalyst characterization

Characterization techniques

Nitrogen (N_2) adsorption-desorption measurements were carried out using an Autosorb IQ-C system cooled to the temperature of liquid N_2 after outgassing the sample under vacuum at 300 °C for 10 h. The specific surface area of the samples was determined using the Brunauer-Emmett-Teller (BET) method. X-ray diffraction (XRD) patterns of materials were recorded on a PANalytical XPert Pro powder diffractometer (at 45 kV and 40 mA) using a Cu Kα radiation source (λ = 1.5405 Å) in a 2θ range from 5° to 80°. Small angle XRD data was collected on an Anton Paar SAXSPoint 2.0 system using a copper microfocus X-ray source and a Dectris Eiger 1M detector. Data was collected for one minute under vacuum at a sample to detector distance of 0.575 m and then reduced to a 1D pattern by radially averaging the 2D detector image. High-resolution transmission electron microscopy (HRTEM) analysis was performed using a Tecnai G2 F20 microscope operated at 300 kV. For HRTEM analysis, samples were dispersed in ethanol under sonication, then dropped onto a carbon-coated copper grid for evaporation. H_2 temperature programmed reduction (H_2-TPR) was performed in a Micromeritics AutoChem 2920 instrument. ~50 mg sample was first pre-treated under pure Ar at 300 °C for 1 h to clean the catalyst surface, and then cooled down to 30 °C. After that, H_2 reduction of the as-prepared catalyst was carried out by heating the sample to 900 °C at 10 °C/min in a 10 vol.% H_2/Argon flow (at 30 mL/min). The consumed H_2 of the sample during reduction was monitored by a thermal conductivity detector (TCD). X-ray photoelectron spectroscopy (XPS) was used to evaluate both the chemical composition and oxidation state of the catalyst surfaces. Photoelectron spectra were recorded with a Thermo Scientific ESCALAB 250Xi spectrometer equipped with Al-Kα radiation (hv = 1486.8 eV). The corresponding binding energies were calibrated with the C 1s line at 284.8 eV as a reference. Unless otherwise specified (e.g., as-prepared catalysts and H_2 plasma-treated catalysts), all the supported Ni catalysts (Ni/SiO_2, Ni/MCM-in, Ni/MCM-both and Ni/MCM-out) mentioned in this paper refer to the reduced catalysts (fresh catalysts after reduction and before reaction).

Table S1. The state of the catalysts used in different characterization methods.

Catalyst state	Metal site state	Characterization methods
As-prepared (-a)	NiO sites	XRD, H_2-TPR,
Reduced	Ni NPs	HRTEM, BET, XRD, FTIR,
H_2 plasma-treated (-p)	Ni/NiO sites	H_2-TPR

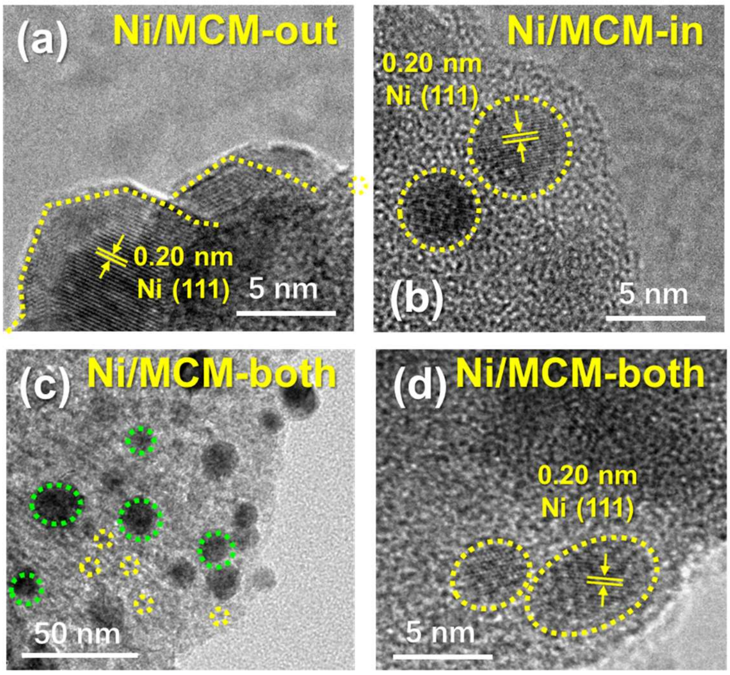

Figure S1. Morphology of local Ni NPs by HRTEM; (a) Ni/MCM-out, (b) Ni/MCM-in, and (c, d) Ni/MCM-both.

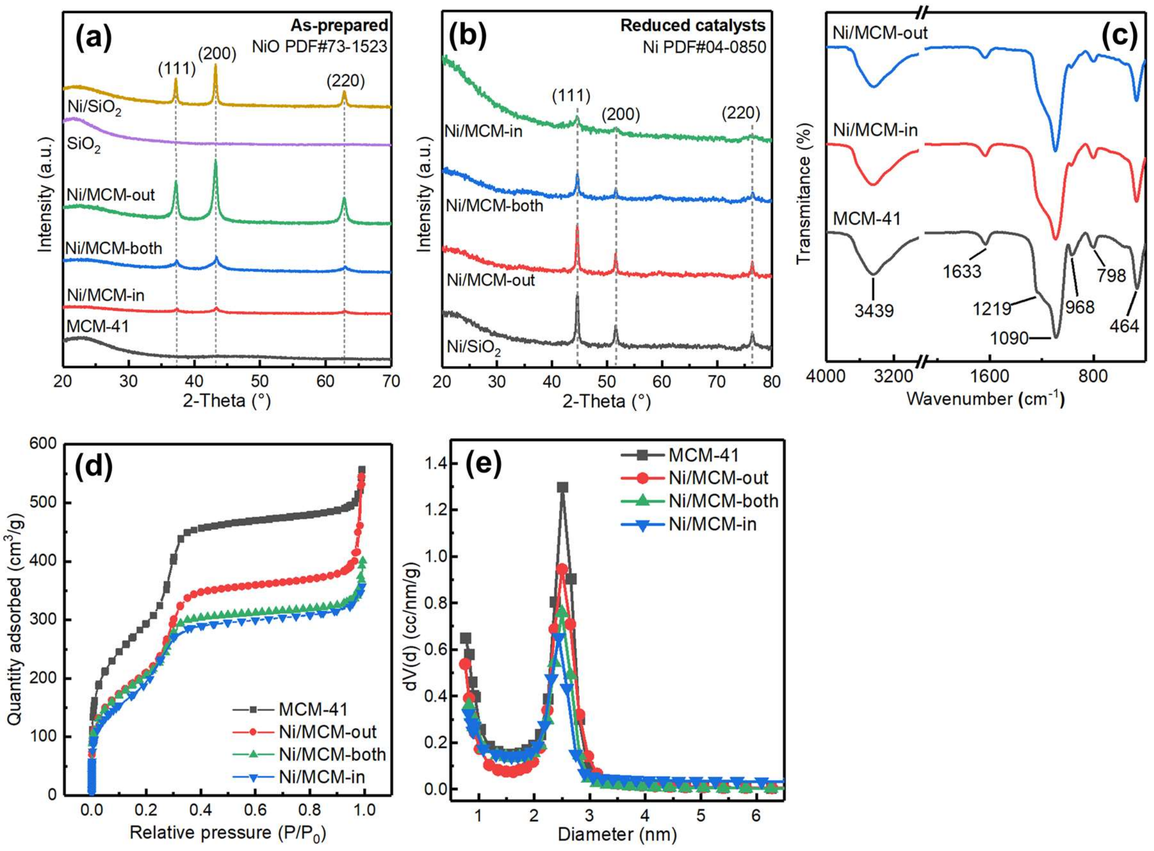

Figure S2. XRD patterns of (a) the as-prepared catalysts and (b) reduced catalysts; (c) FTIR spectra of MCM-41, Ni/MCM-in and Ni/MCM-out; (d) N_2 adsorption-desorption isotherms of the Ni/MCM-41 catalysts; (e) Pore size distribution of the Ni/MCM-41 catalysts.

Table S2. Physical properties of the supports and the reduced catalysts.

	BET surface area (m^2/g)[a]			Average pore size (nm)	Grain size (nm)[b]	Mean particle size (nm)[c]	D_{metal} (%)[d]
	Total	External	Internal				
SiO_2	334	305	29	6.3	-	-	-
Ni/SiO_2	251	223	28	7.3	24	19	2.6
MCM-41	1052	115	984	3.3	-	-	-
Ni/MCM-in	685	221	464	2.4	10	5	9.0
Ni/MCM-both	751	238	513	2.9	15	11	4.7
Ni/MCM-out	757	246	511	3.0	17	15	3.5

[a] Total surface area was determined by the BET method, and internal surface area was determined by the t-plot method; [b] Ni grain size was calculated using the Scherrer equation (eq S1) based on the XRD patterns of the catalysts; [c] Ni NP size was determined by HRTEM; [d] Ni dispersion (D_{metal}) was calculated according to the mean particle size [1].

$$\tau = \frac{K\lambda}{(\beta - \beta_0)\cos\theta} \qquad \text{(S1)}$$

Where τ is the mean grain size; K is the dimensionless shape factor (typically 0.89); λ is the X-ray wavelength of the Cu source; β is the peak width at half peak height, and β_0 is the instrumental line broadening; θ is the X-ray incidence angle with respect to the sample surface. In this work, the grain size was obtained by Jade 6.0.

Comparative XRD patterns of the catalysts under study (Figures S2a and S2b) show the appearance of metallic Ni peaks (Ni PDF# 04-0850) after reduction, which confirms that all Ni species have been sufficiently reduced before performing the plasma-assisted catalysis. FTIR spectra of the Ni/MCM-41 catalysts are the same as that of MCM-41 without the vibration of Ni-Si, which illustrates the limited interaction between metallic Ni and the MCM-41 support (Figure S2c) [2]. The adsorption and desorption isotherms of all the samples (Figure S2d) show that the adsorption is a type IV isotherm with the hysteresis loop, this is consistent with previous findings showing the presence of a well-ordered mesoporous structure in these catalysts [3]. The pore size distribution of MCM-41 and Ni/MCM-41 confirms the presence of uniform mesopores in these samples with a diameter of about 2.5 nm, and the loading of Ni within MCM-41 (i.e., Ni/MCM-in) caused a slightly narrower distribution of mesopores, which centred at about 2.3 nm, as shown in Figure S2e.

In this work, the nickel dispersion (D_{metal}) was calculated using the particle size (d) using eq S2 [1].

$$D_{metal} = \frac{5\times10^{10}\rho_s W_m}{N_a \rho_m d} \qquad \text{(S2)}$$

where ρ_s is the surface sites density and is considered constant (1.54 for Ni), W_m is the molecular weight of the metal, ρ_m is the atomic density and N_a is Avogadro's number.

The ammonia synthesis rate (R_{NH_3}) is calculated as the ratio of moles of ammonia to the amount of catalyst used in the performance tests (eq S3). To make the performance more comparable, the amount of catalyst under "plasma alone" condition is assumed to be 0.5 g, which is the same amount as the catalysts used in other tests.

$$R_{NH_3}\,(\mu mol/g/h) = \frac{\text{Number of moles of produced ammonia } (\mu mol\ h^{-1})}{\text{The amount of catalyst used (g)}} \quad (S3)$$

Further, the turnover frequency (TOF) is defined as the ratio of moles of ammonia to moles of the exposed Ni NPs of the catalysts (eq S4). The moles of the exposed metal sites were calculated by multiplying the total moles of Ni loading with the metal dispersion (D_{metal}). D_{metal} was calculated using the average particle sizes of Ni NPs of different catalysts, which were determined by HRTEM analysis [4]. Note that chemisorption was performed on the catalysts under investigation. However, useful information on Ni dispersion could not be achieved.

$$\text{TOF}\left(s^{-1}\right) = \frac{\text{Number of moles of produced ammonia (mol } s^{-1})}{D_{metal}\ (\%) \times \text{Number of moles of metal loading (mol)}} \quad (S4)$$

Detailed procedures for the H_2-plasma treatment of samples

Step 1. The catalyst powder with 0.5 g, including Ni/SiO_2, Ni/MCM-out/both/in, was loosely filled into the DBD reactor (which was consistent with the one used for the plasma-assisted reaction).

Step 2. Then, pure hydrogen with 60 mL/min was introduced to purge the whole system for 30 min in order to remove the residual air, and the composition of the outlet gas was determined by a Jasco FT/IR-4600 FTIR spectrometer.

Step 3. Subsequently, the H_2 flow rate was lowered to 40 mL/min, and then, the hydrogen plasma was ignited at 36 kJ/L (9.2 kHz) for 3 h. This stage can be considered as the catalyst reduction process by plasma.

Step 4. After the discharge, the plasma-treated catalysts were collected from the reactor and stored into a sample bottle filled with argon, followed by H_2-TPR tests. This step can efficiently prevent the reoxidation of Ni NPs over the catalyst surfaces.

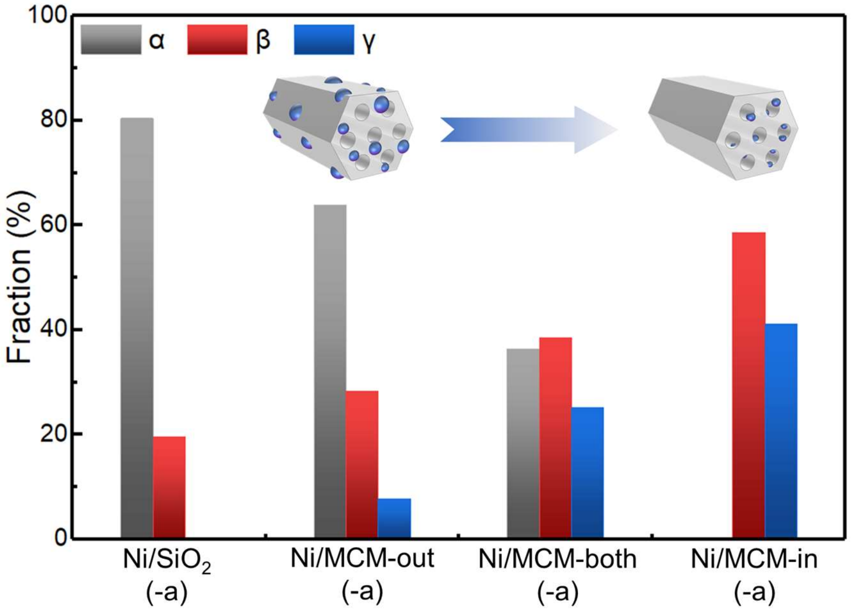

Figure S3. Proportions of different Ni sites in the as-prepared catalysts under investigation according to H_2-TPR.

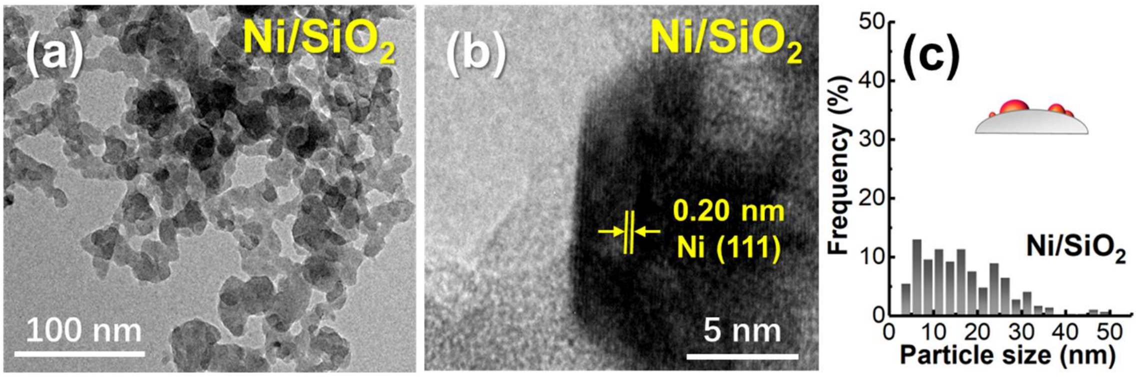

Figure S4. (a) and (b) HRTEM images and (c) particle size distribution of the control catalyst (Ni/SiO_2).

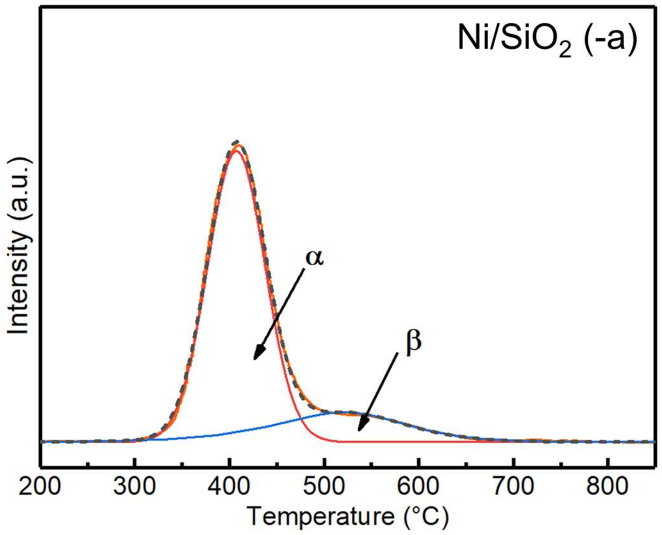

Figure S5. H_2-TPR profile of the as-prepared control catalyst of Ni/SiO_2 (-a).

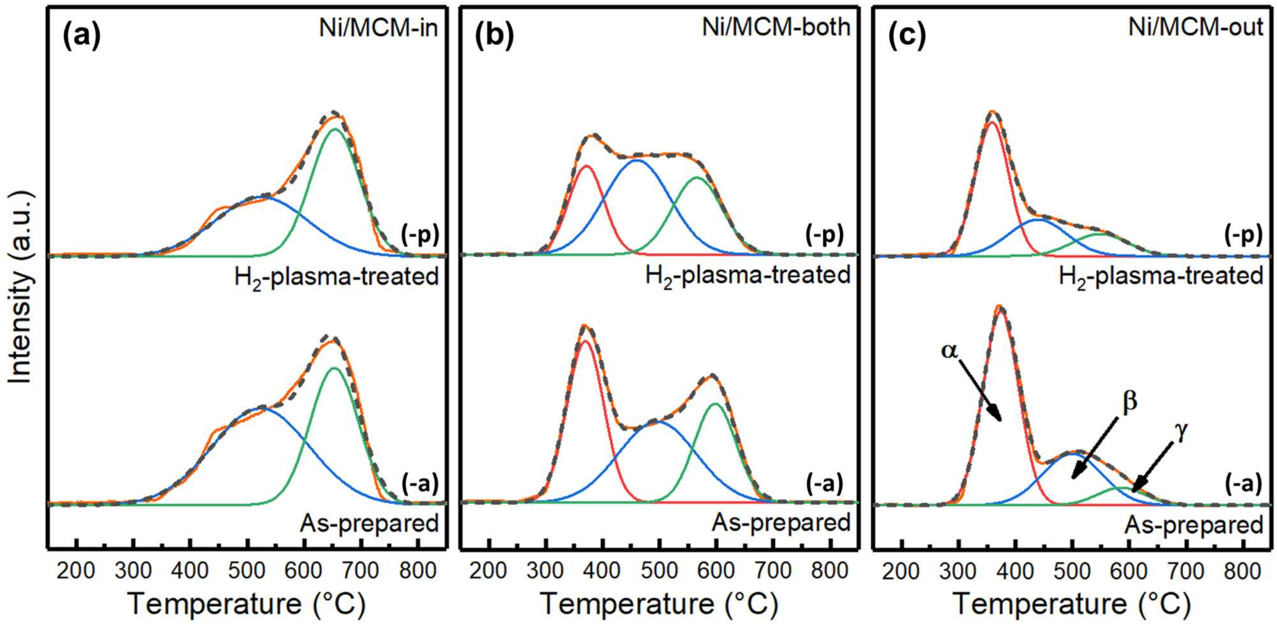

Figure S6. H_2-TPR profiles of the as-prepared (-a) and H_2 plasma-treated catalysts (-p). (a) Ni/MCM-in, (b) Ni/MCM-both, (c) Ni/MCM-out. (H_2 plasma treatment conditions: at 36 kJ/L and 40 mL/min with 100 vol. % H_2 for 3 h for each catalyst).

Table S3. Peak information of H_2-TPR of the as-prepared catalysts (-a) before and after the H_2 plasma treatment (-p).

	Peak temperature (°C)			H_2 consumption (μmol/g)			
	α	β	γ	α	β	γ	Total
Ni/MCM-in (-a)	-	524	653	-	37.0	26.1	63.1
Ni/MCM-in (-p)	-	527	657	-	24.0	27.4	51.4
Ni/MCM-both (-a)	370	496	598	23.9	23.1	15.1	62.1
Ni/MCM-both (-p)	372	460	566	9.2	18.1	12.2	39.5
Ni/MCM-out (-a)	374	500	586	42.1	18.7	5.1	65.9
Ni/MCM-out (-p)	359	439	548	20.7	9.7	5.6	35.9
Ni/SiO_2 (-a)	407	521	-	53.2	13.0	-	66.2

2. Electrical signal analysis and plasma diagnostics

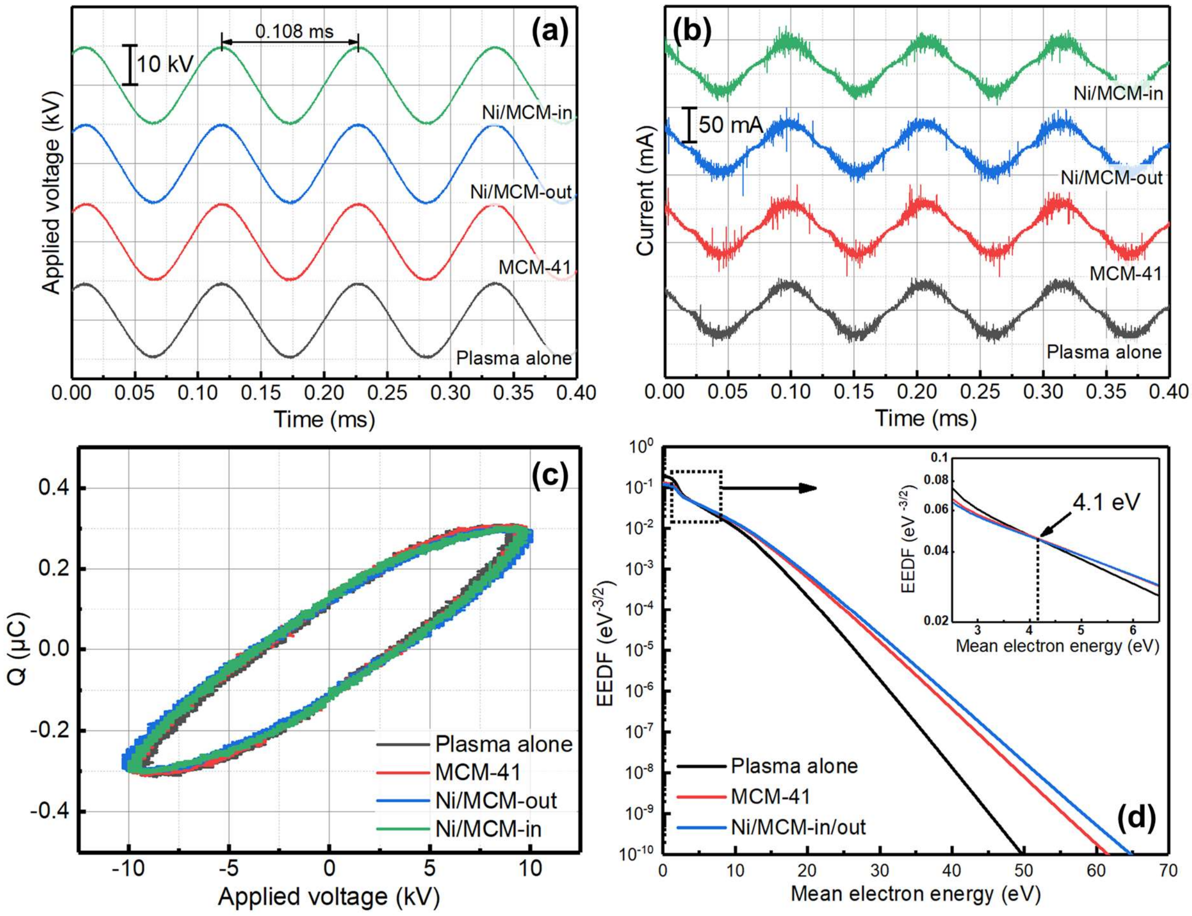

Figure S7. (a) Applied voltage signals, (b) current signals, (c) Lissajous plots and (d) Maxwellian EEDF in $eV^{-3/2}$ for mean electron energies from 0–70 eV in the plasma alone and plasma-catalytic systems (SEI = 24 kJ/L).

Table S4. Discharge parameters of the plasma alone and plasma-catalytic systems.[a]

	U_{pp}[b] (kV)	C_{cell}[b, c] (μF)	C_{diel}[b, c] (μF)	U_b[b] (kV)	n_e[b] ($\times 10^{17}$, m^{-3})
Plasma alone	18.6±0.4	4.9±0.5	34.6±1.0	2.8±0.1	6.53
MCM-41	19.2±0.2	5.1±0.5	33.3±1.0	3.0±0.1	5.62
Ni/MCM-in	19.6±0.2	4.7±0.5	32.2±1.0	3.2±0.1	5.53
Ni/MCM-out	19.8±0.2	4.3±0.5	31.7±1.0	3.2±0.1	5.37

[a] Discharge parameters were calculated based on Figure S7c at 24 kJ/L; [b] U_{pp}: peak-to-peak applied voltage; C_{cell}: cell capacitance; C_{diel}: dielectric capacitance (quartz tube in this work); U_b: breakdown voltage; n_e: electron density; [c] Both C_{cell} and C_{diel} can be determined by Lissajous plots in Figure S7c. [5–7]

C_g (the capacitance of the gap) and U_b were determined according to eq S5 and eq S6, respectively.

$$C_g(\mu F) = \frac{C_{diel}(\mu F) \times C_{cell}(\mu F)}{C_{diel}(\mu F) - C_{cell}(\mu F)} \quad \text{(S5)}$$

$$U_b(kV) = \frac{1}{1 + \left(C_g(\mu F)/C_{diel}(\mu F)\right)} \times U_{min}(kV) \quad \text{(S6)}$$

where U_{min} can be determined from the Lissajous figures (Figure S7c). U_{min} refers to the minimum external voltage at which the plasma ignition occurs [5–7].

3. Calculation of electron density and Debye length

The mean electron density (n_e) can be determined by eq S7: [8,9]

$$n_e\left(\mathrm{m}^{-3}\right)=\frac{J\ (\mathrm{A\ m}^{-2})}{E/N(\mathrm{Td})\times\mu_e\times e} \tag{S7}$$

where J is the current density, defined as the ratio of the average peak discharge current over 3 cycles to the surface area of one micro-discharge (1.05×10^{-6} m^2) [10]; E is the average electric field; μ_e is the electron mobility calculated from BOLSIG+; e is the electron charge.

The Debye length (λ_D) can be determined by:

$$\lambda_D=\sqrt{\frac{\varepsilon_0\kappa T_e}{n_e q_e^2}} \tag{S8}$$

where ε_0 is the permittivity of free space; κ is Boltzmann's constant; T_e is the temperature of electrons (T_e can be obtained via the transformation of E_e); q_e is the charge on an electron. eq S8 is valid when the ions are much colder than the electrons in the plasma.

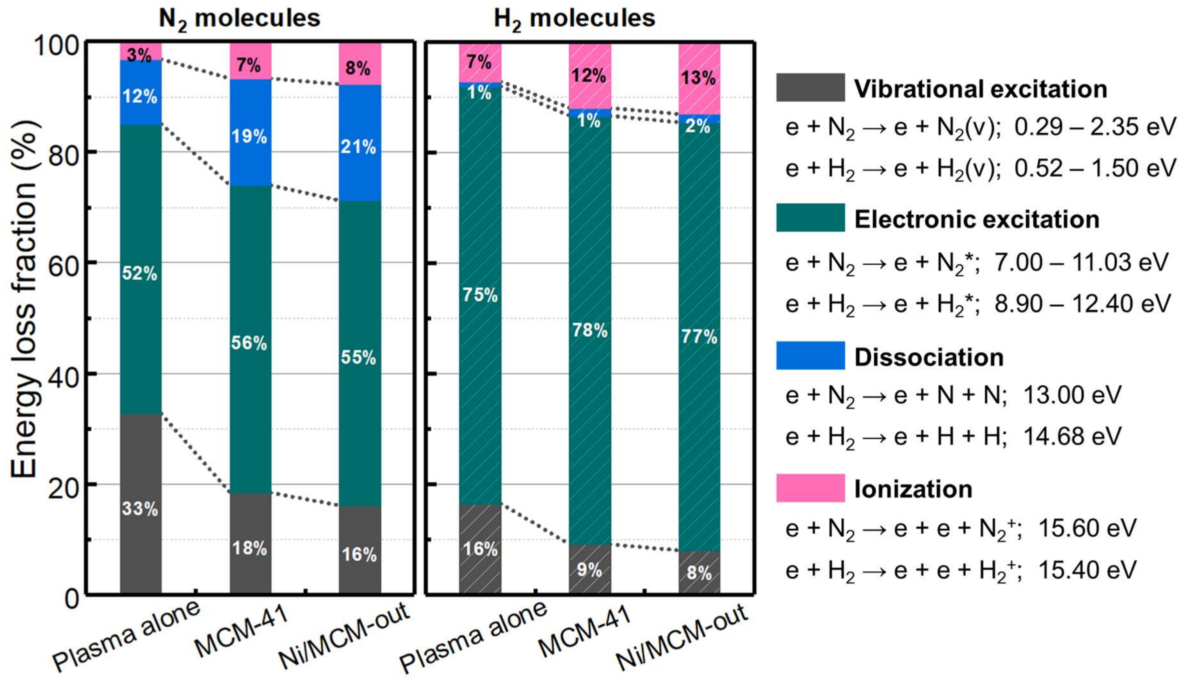

Figure S8. Energy loss fractions of N_2 and H_2 molecules determined by BOLSIG+. The energy thresholds of four electron-induced reaction channels for N_2 and H_2 (on the right) are concluded from the open-access LXCat dataset (www.lxcat.net).

4. TOF vs external Ni sites

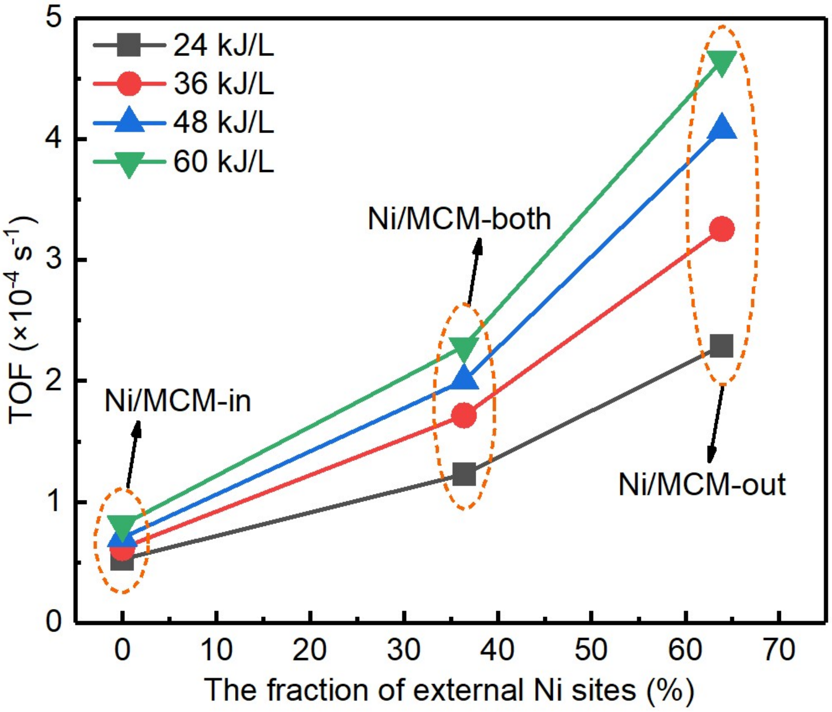

Figure S9. The TOF values vs the fraction of external Ni sites on Ni/MCM-41 catalysts

5. Stability tests of Ni/MCM-out

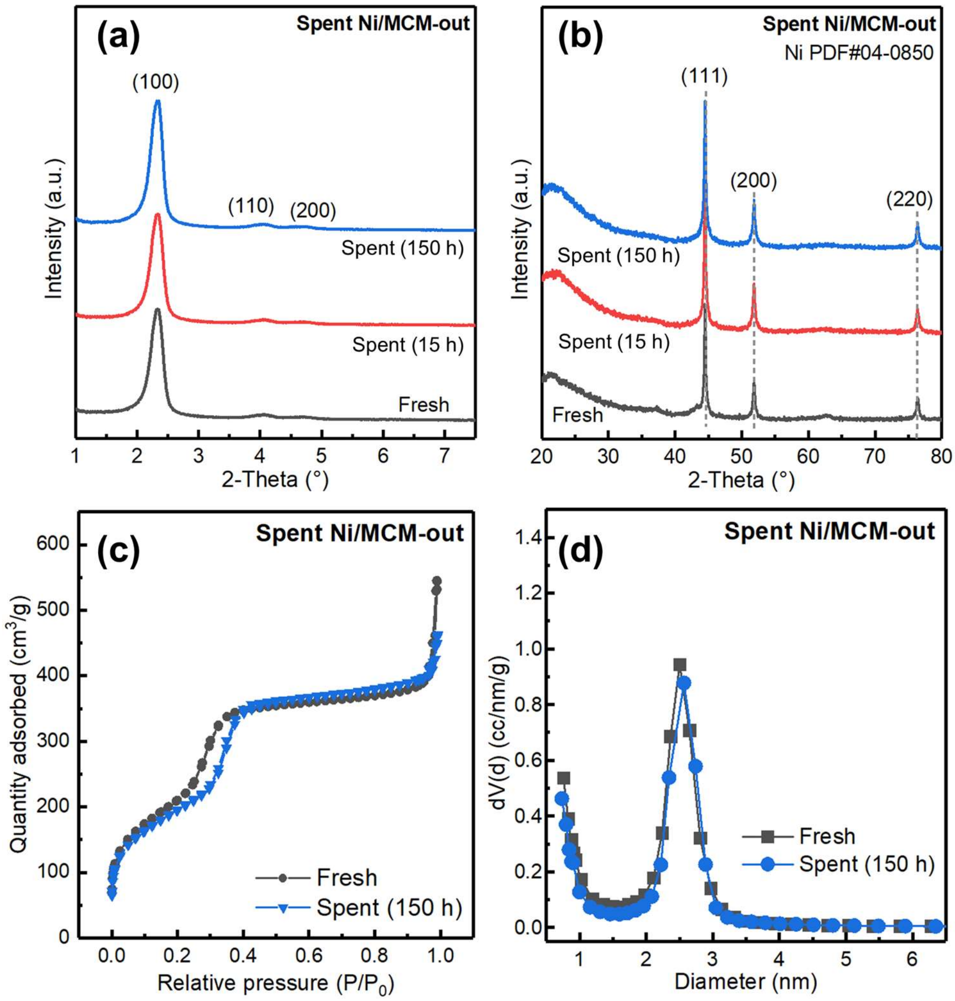

Figure S10. (a) Small-angle and (b) high-angle XRD patterns of the fresh Ni/MCM-out catalyst and the spent catalysts after the 15 h and 150 h plasma-catalytic NH_3 synthesis; (c) N_2 adsorption-desorption isotherms and (d) pore size distribution of the fresh Ni/MCM-out catalyst and the spent catalyst after the 150 h plasma-catalytic NH_3 synthesis.

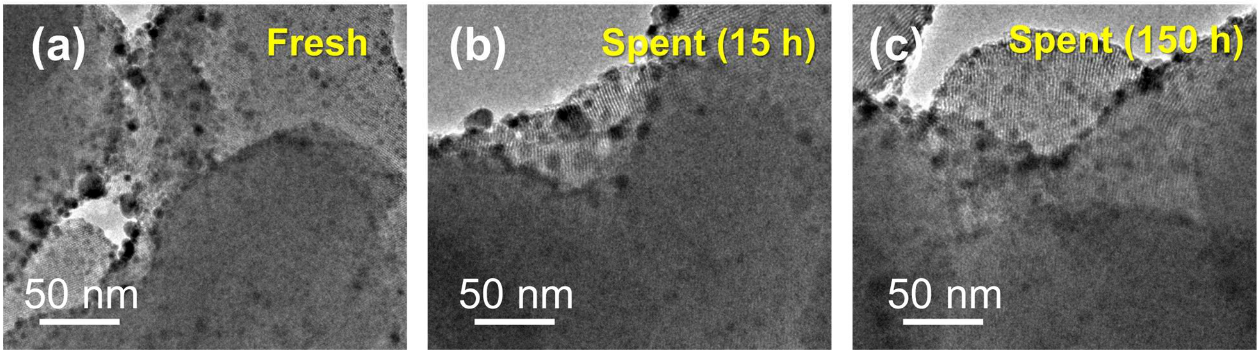

Figure S11. HRTEM images of (a) the fresh Ni/MCM-out catalyst, and the spent catalysts after (b) 15 h and (c) 150 h plasma-catalytic NH_3 synthesis.

Table S5. Comparison of specific surface area between the fresh and spent Ni/MCM-out catalysts.

	BET surface area (m^2/g)			Average pore size (nm)
	Total	External	Internal	
Fresh Ni/MCM-out	757	246	511	3.0
Spent Ni/MCM-out[a]	782	262	520	2.7

[a] after 150 h longevity test.

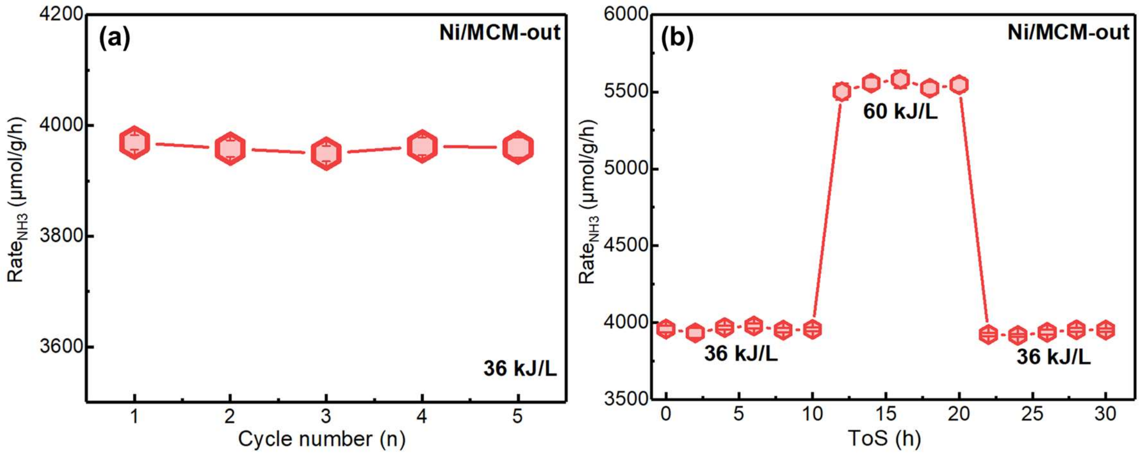

Figure S12. (a) Cyclic stability tests of Ni/MCM-out under plasma conditions for NH_3 synthesis (Q_{gas} = 40 mL/min, at 35 °C and 1 bar). Each experiment (cycle) lasted 4 h, and Ar (40 mL/min) was used to purge the system overnight between each test. The SEI for each cycle was maintained at 36 kJ/L. (b) R_{NH_3} over Ni/MCM-out as a function of time-on-stream (ToS) at different SEIs. (Errors were determined by the continuous measurements within 5 min at each sampling point).

6. Energy efficiency of plasma synthesis of ammonia using different catalysts

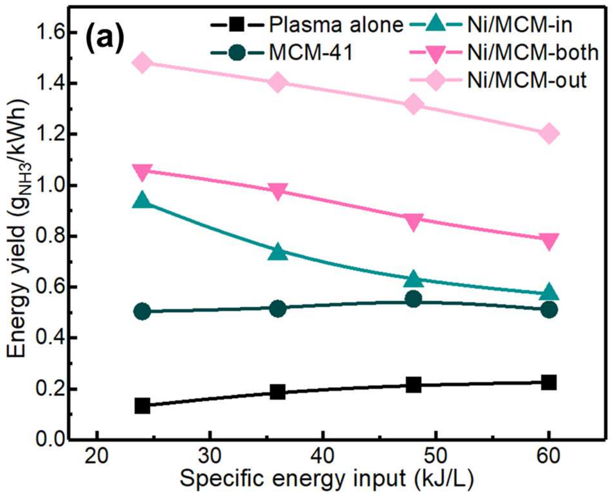

Figure S13. (a) Effect of SEI and catalysts on the energy yield of ammonia at different discharge powers. (Q_{gas} = 40 mL/min, Discharge power: 8–40 W, at 35 °C and 1 bar).

7. High flow rate tests of Ni/MCM-out

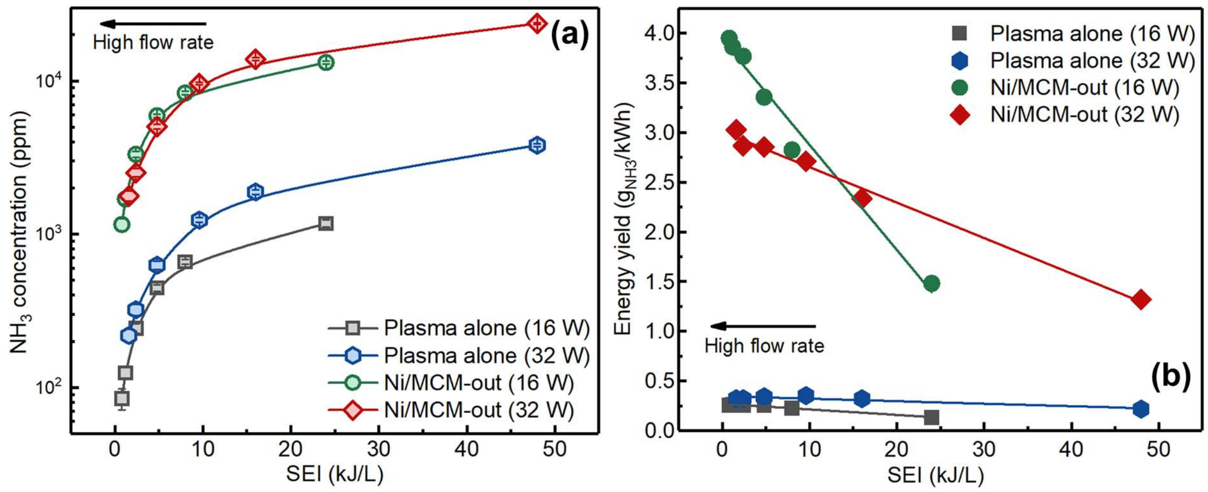

Figure S14. Effect of SEI on (a) NH_3 concentration and (b) energy yield using different flow rates. (Q_{gas} = 40–1200 mL/min, at 35 °C and 1 bar). Each experiment lasted 3 h. The discharge power was fixed at 16 W and 32 W, respectively.

8. Literature comparison of plasma-assisted ammonia synthesis

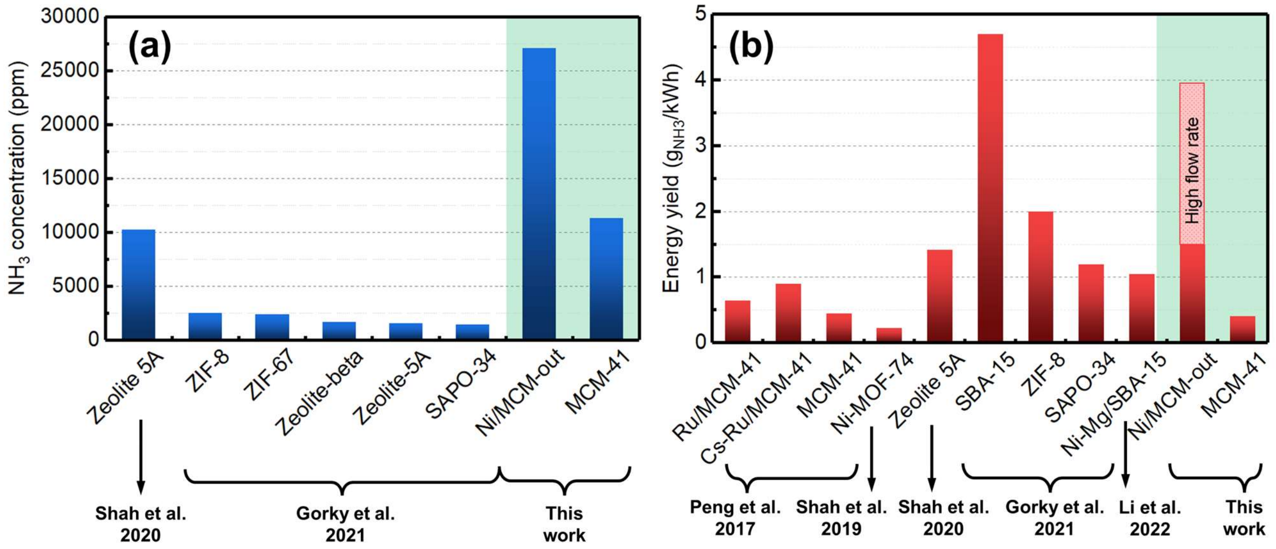

Figure S15. Reported (a) NH_3 concentration and (b) energy yield from the plasma systems employing porous materials (including zeolites and MOFs). Plotted data is obtained from the works of Shah et al[11,12] (Ni-MOF-74 was tested at 0.7 Torr.), Gorky et al[13,14], Peng et al[15], Li et al[16].

Table S6. Comparison of the current state-of-the-art performance in the plasma-assisted ammonia synthesis using DBD at atmospheric pressure.

Catalyst	Discharge parameters			Reaction conditions		Performance		Energy yield	Ref
	f (kHz)	U_p (kV)	P_d (W)	N_2/H_2	Q_{gas} [b] (mL/min)	Conc. [d] (ppm)	Yield (%)	g_{NH3}/kWh	
Ru/Alumina membrane	21.5		127	1:3	30	26611	4.62	0.4	17
$Pb[Zr_xTi_{1-x}]O_3$ [a]	0.5	2.5		1:3	11.5			0.44	18
Cs-10Ru/MgO	8	5		3:1	4000		3.6		19
$Pb[Zr_xTi_{1-x}]O_3$ [a]	1	2.5		1:3	11.5		4.7	0.26	20
$Pb[Zr_xTi_{1-x}]O_3$ [a]	5	4.1		1:3	38.3		4	0.41	20
$2Ru/Al_2O_3$			7	4:1	2000	220	0.014	2.86	21
$2Ru/Al_2O_3$			22	4:1	2000	850	0.053	3.52	21
$2Ru\text{-}5Mg/Al_2O_3$			7	4:1	2000	1680	0.105	21.86	21
Ru/MCM-41				1:1				0.65	15
Cs-Ru/MCM-41				1:1				0.9	15
Ni/SiO_2	20		140	1:3	25	28320	5.9	0.44	22
$BaTiO_3$	20		93	1:3	25	18240	3.8	0.43	22
$Ni/SiO_2\text{-}BaTiO_2$	20		115	1:3	25	30720	6.4	0.57	22
Au-wool	50	5		1:3	100	25641	2.5		23
Ni/Al_2O_3			10	1:2	100	4000	2	0.89	24
$10Ni/Al_2O_3$			55	1:2	40	6324	0.9	0.29	25

$10Cu/Al_2O_3$			55	1:2	40	6203	0.9	0.29	[25]
$10Fe/Al_2O_3$			55	1:2	40	5499	0.8	0.25	[25]
$5Co/Al_2O_3$	20-25		10	3:1	50		2.6		[26]
$5Ni/Al_2O_3$	20-25		10	3:1	50		2.1		[26]
$5Ru/Al_2O_3$	20-25		10	3:1	50		1.5		[26]
5Ru/L-MgO	9.2		60	2:1	300	2689	4	1.29	[27]
$5Ru/Al_2O_3$	9.2		60	2:1	300	2165	3.2	1.04	[27]
$5Fe/Al_2O_3$	18.3	0.42	1	2:1	22.5 (20 He) [c]	1792	0.17		[28]
$5Ni/Al_2O_3$	18.3	0.42	1	2:1	22.5 (20 He) [c]	1892	0.18		[28]
Al_2O_3	18.3	0.42	1	2:1	22.5 (20 He) [c]	1394	0.03		[28]
$5Ni/Al_2O_3$			10	2:1	40		1.4		[29]
$5Pt/Al_2O_3$			10	2:1	40		1.6		[29]
$10Ni/Al_2O_3$			42	2:1	180	4600			[30]
$10Co/Al_2O_3$	21		42	2:1	180	5800			[30]
$10Mo/Al_2O_3$	21		42	2:1	180	5750			[30]
$10W/Al_2O_3$	21		42	2:1	180	5000			[30]
$2Ru/Al_2O_3$	21		42	2:1	180	6300			[30]
$2Pt/Al_2O_3$	21		42	2:1	180	6200			[30]
Zeolite-5A			20	1:3	25	9081	1.8	0.513	[11]
Alkaline-Al_2O_3			10	1:3	50	2100	0.42	0.48	[31]
α-Al_2O_3	21		46.7	1:3	400	2600	0.52	1.01	[32]
γ-Al_2O_3	21		46.7	1:3	400	2700	0.54	1.05	[32]
MgO	21		46.7	1:3	400	2400	0.48	0.94	[32]
CaO	21		46.7	1:3	400	2300	0.46	0.9	[32]
$5Ru/Al_2O_3$	20	9	50	1:3	120	7800	1.5	1.27	[33]
$5Ru/Al_2O_3$	20	9	38.4	2:1	120	14900	1.1	1.88	[33]
$5Ni/TiO_2$	50	6		1:1	100	10080	1		[34]
$5Ni/Al_2O_3$	50	6		1:1	100	24640	2.4		[34]
$5Fe/Al_2O_3$	50	6		1:1	100	6720	0.67		[34]
$5Ru/Al_2O_3$	50	6		1:1	100	2912	0.29		[34]
TiO_2	50	6		1:1	100	7324.8	0.7		[34]
Al_2O_3	50	6		1:1	100	12320	1.2		[34]
ZIF-8				1:3	25	2555.4			[13]
ZIF-67				1:3	25	2387.8			[13]
Zeolite-beta				1:3	25	1700			[13]
Zeolite-5A				1:3	25	1612			[13]
SAPO-34				1:3	25	1478			[13]
Ru/MgO			3.8	1:1	20	5000			[35]
Ru-K/MgO			3.8	1:1	20	6100			[35]
SBA-15	20-23	8.6	15	1:3	25	6120		4.6	[14]
ZIF-8	20-23	8.6	15	1:3	25	2580		1.9	[14]
Fumed-SiO_2	20-23	8.6	15	1:3	25	1790		1.4	[14]
SAPO-34	20-23	8.6	15	1:3	25	1610		1.2	[14]

Non-porous SiO_2	20-23	8.6	15	1:3	25	1345		1.1	14
5Co/Carbon	10.1		21.3	1:3	100	2880	0.57	0.63	36
5Ru/Carbon	10.1		21.3	1:3	100	3000	0.6	0.66	36
Ni-$Mg_{0.02}$/SBA-15-IWI [e]	8	9		1:1	20			1.05	16
MCM-41	9.2	9.4	40	1:3	40	11345	2.2	0.5	This work
Ni/MCM-out	9.2	9.7	40	1:3	40	27115	5.3	1.2	
Ni/MCM-out	9.2	7.8	16	1:3	40	13162	2.6	1.5	
Ni/MCM-out	9.2	7.8	16	1:3	1200	1155	0.2	3.9	

[a] *x*: the weight loading of Zr; [b] Total flow rate; [c] He: 20 vol. %; [d] Conc.: concentration of NH_3; [e] IWI: incipient wetness impregnation.

9. In situ FTIR characterization of the catalyst surface under plasma discharge

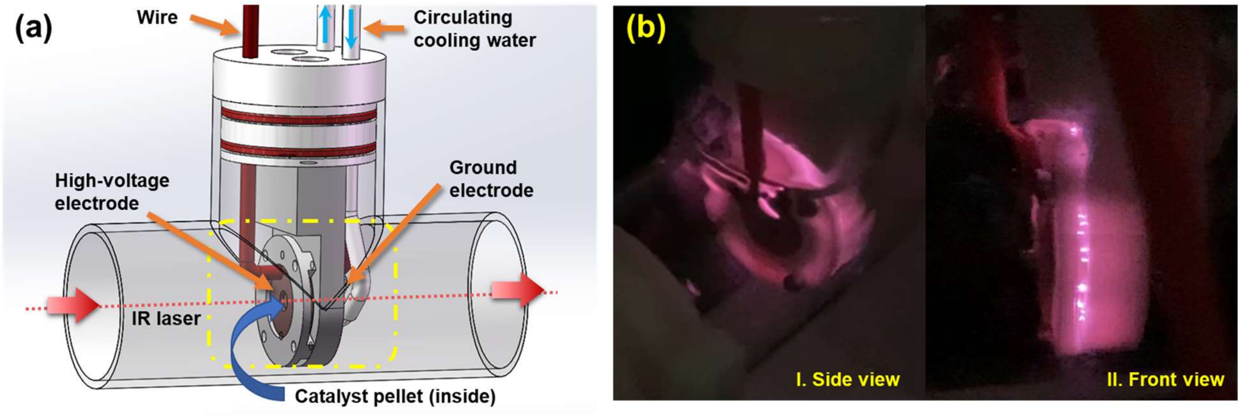

Figure S16. (a) Schematic and (b) experimental photos of the home-designed in situ transmission FTIR gas cell for the plasma-catalytic reactions.

For in situ FTIR analysis, three experimental procedures (A, B and C) were designed to investigate 3 different processes in the plasma-catalytic ammonia synthesis, as detailed below.

A. Adsorption of N_2 on the catalysts in N_2 plasma

Step 1. Prior to the in situ FTIR measurements, the catalyst was pre-treated by Ar plasma (99.999% purity, at 100 mL/min) in the DBD reactor at 90 °C for 20 min to clear its surface.

Step 2. N_2 (99.999% purity, at 40 mL/min) was used to flush the gas cell for 30 min to displace Ar. During this process, the temperature was cooled from 90 °C to 35 °C. After that, the IR background was collected at 35 °C.

Step 3. After switching off the gas, the plasma was switched on (at discharge power of ~6 W), and the IR spectra were collected every 2 min for 10 min.

Step 4. Finally, the plasma was switched off, and Ar was used to flush the gas cell after the reaction. The IR spectra were collected continuously for 30 min with the decrease of adsorbed IR peaks.

B. Hydrogenation of the adsorbed N_2 in H_2 plasma

Step 1. Prior to the in situ FTIR measurements, the catalyst was pre-treated by Ar plasma (99.999% purity, at 100 mL/min) in the DBD reactor at 90 °C for 20 min to clear its surface.

Step 2. N_2 (99.999% purity, at 40 mL/min) was used to flush the gas cell for 30 min to displace Ar. During this process, the temperature was cooled from 90 °C to 35 °C. Then, N_2 was switched off and H_2 (99.999% purity, at 40 mL/min) was introduced for 10 min to replace the remaining N_2 in the cell. After that, the IR background was collected at 35 °C.

Step 3. After switching off the gas, the plasma was switched on (at discharge power of ~6 W), and the IR spectra were collected every 2 min for 10 min.

Step 4. Finally, the plasma was switched off, and Ar was used to flush the gas cell after the reaction. The IR spectra were collected continuously for 30 min with the decrease of adsorbed IR peaks.

C. Plasma-assisted NH_3 synthesis in N_2-H_2 plasma

Step 1. Prior to the in situ FTIR measurements, the catalyst was pre-treated by Ar plasma (99.999% purity, at 100 mL/min) in the DBD reactor at 90 °C for 20 min to clear its surface.

Step 2. A mixture of N_2 and H_2 (N_2/H_2 = 1:3; total flow rate: 40 mL/min) was introduced to flush the cell for 30 min. During this process, the temperature was cooled from 90 °C to 35 °C. After that, the IR background was collected.

Step 3. After switching off the gas, the plasma was switched on (at discharge power of ~6 W), and the IR spectra were collected every 2 min for 10 min.

Step 4. Finally, the plasma was switched off, and Ar was used to flush the cell after the reaction. The IR spectra were collected continuously for 30 min with the decrease of adsorbed IR peaks.

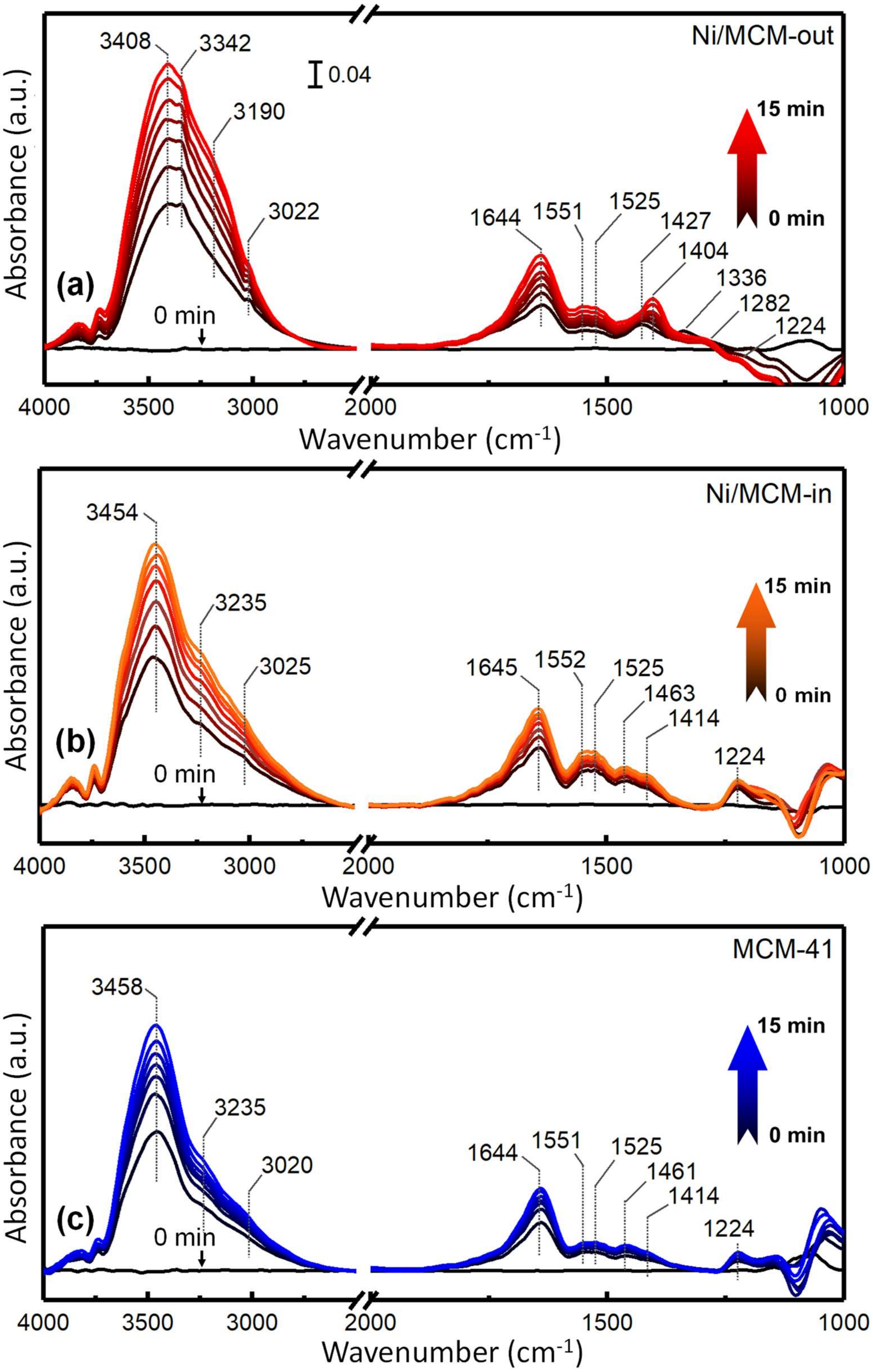

Figure S17. In situ FTIR spectra of plasma-driven ammonia synthesis on (a) Ni/MCM-out, (b) Ni/MCM-in and (c) MCM-41, respectively. (Q_{gas} = 40 mL/min, discharge time = 15 min, SEI = 10 kJ/L, N_2/H_2 = 1:3, 1 bar. Each IR spectrum was collected every 3 min.)

Table S7. Infrared bands information of the surface adsorbed species for the plasma-catalytic synthesis of NH_3 at 35 °C and 1 bar).

Surface species	Wavenumber (cm^{-1})	Assignment	Literature value (cm^{-1})
Coordinated NH_3 $*NH_3$ or $*NH_4^+$	3458, 3454, 3408, 3393, 3342	ν_{as} (NH_3)	3272[37], 3340[37], 3393[37], 3443[37], 3324[38], 3405[38]
	3235, 3222, 3190	ν_s (NH_3)	3250[38]
	1645, 1644, 1639	$\delta_{as}(NH_3)$	1624[38]
	1552, 1551, 1550, 1525	$\delta_{as}(NH_3)$ or $\delta_{as}(NH_4^+)$	1593[37], 1550[39]
	1224	$\delta_s(NH_3)$	1260–1300[38], 1297[37], 1120–1250[39]
(with different coordinative unsaturation)	1336, 1282	$\delta_s(NH_3)$	1267–1280[38], 1278–1291[38]
NH_x (1-2) amide groups $*NH$ or $*NH_2$	3120	ν_{as} (NH_x)	3170[37]
	3022, 3025, 3020, 3019	ν_s (NH_x)	3004[38], 3020[38]
	1463, 1461	H-N-H bending of NH_x species	1475[39], 1444[40], 1450[40]
	1427, 1414, 1410, 1404, 1403	NH deformation	1400–1450[41], 1445[39]
Adsorbed N_2 molecules $*N_2$	1935	N=N stretching or ν(NN)	1908[42], 1913[42], 1942[42], 1858–1991[42], 1934[43,44], 1990–2090[45,46]

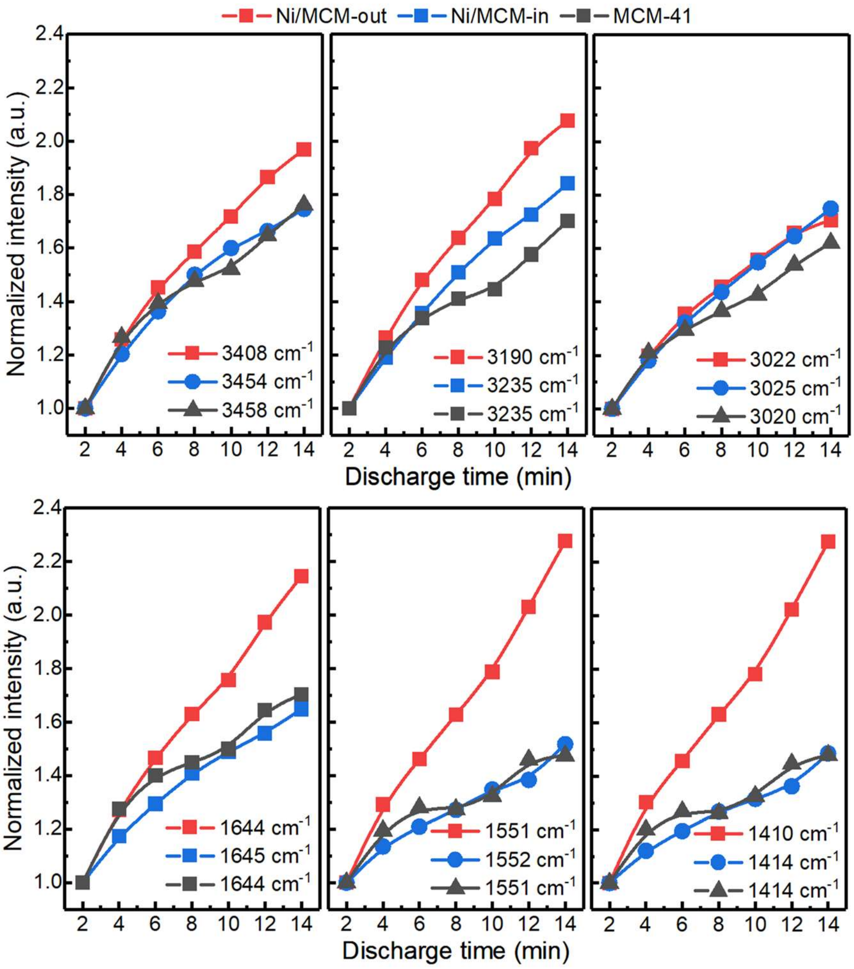

Figure S18. Normalized IR band intensities as a function of discharge time to show the evolution of surface species on MCM-41 and Ni/MCM-in/out under N_2-H_2 plasma condition at room temperature. (*NH_3: 3408, 3454, 3458 (ν_{as} (NH_3)), 3190, 3235 (ν_s (NH_3)), 1644, 1645 δ_{as}(NH_3); *NH_x: 3022, 3025, 3020 ν_s (NH_x); *NH: 1414, 1410 NH deformation).

The IR band intensities of the correspongding IR peaks were normalized by comparing the intensity values at different discharge times and the intensity at the initial 2 min discharge, as shown in the following equation.

$$\text{Normalized IR intensity} = \frac{\text{Peak intensity at } x \text{ min discharge } (2 < x < 14 \text{ min})}{\text{Peak intensity at 2 min discharge}} \quad \text{(S9)}$$

Table S8. Distribution of NH_x* (x = 0, 1, 2 and 3) surface species on the spent MCM-41, Ni/MCM-in and MCM-out according to XPS analysis. [25]

	NH_3*	NH_2*	NH*	N*
N1s BE (eV)	400.7	399.5	398.7	397.6
MCM-41[a]	67%	17%	11%	5%
Ni/MCM-in	28%	32%	26%	14%
Ni/MCM-out	23%	34%	24%	19%

[a] The fraction for each species is normalized by the peak areas for each catalyst in N 1s XPS spectra (Figure 3d).

10. Supplementary results of OES diagnostics

Corresponding to the IR spectra above, optical emission spectroscopic (OES) was measured to better understand the presence of reactive species in the gas phase of N_2-H_2 plasma over MCM-41, Ni/MCM-in, and Ni/MCM-out, respectively. The plasma alone was used as the control group.

Figure S19 shows the schematic of the experimental rig for the OES diagnostics. The operating parameters were kept the same as that used in the plasma-assisted reaction with a total flow rate of 40 mL/min (N_2/H_2 = 1:3) at 8 W (9.2 kHz). The emission spectra were recorded when the plasma reached a steady state. For the spectra with full-ranged wavelength (200 – 800 nm, shown in Figure S20a), the optical grating, and exposure time were 600 g/mm and 600 ms, respectively. For the zoomed-in view of the emission peaks of NH, N and H_α, the optical grating and exposure time were 2400 g/mm and 30 s, respectively.

To compare the relative intensity of key atomic lines (N and H_α) and molecular band (NH) in the spectra, their intensities were firstly normalized against the intensity of the (0, 0) band head of N_2 second positive system, which was assigned the value 1. After that, the obtained normalized intensity of each line under three different conditions (plasma alone, plasma with MCM-41, plasma with Ni/MCM-in and plasma with Ni/MCM-out) were normalized again using the method reported in our previous work [25].

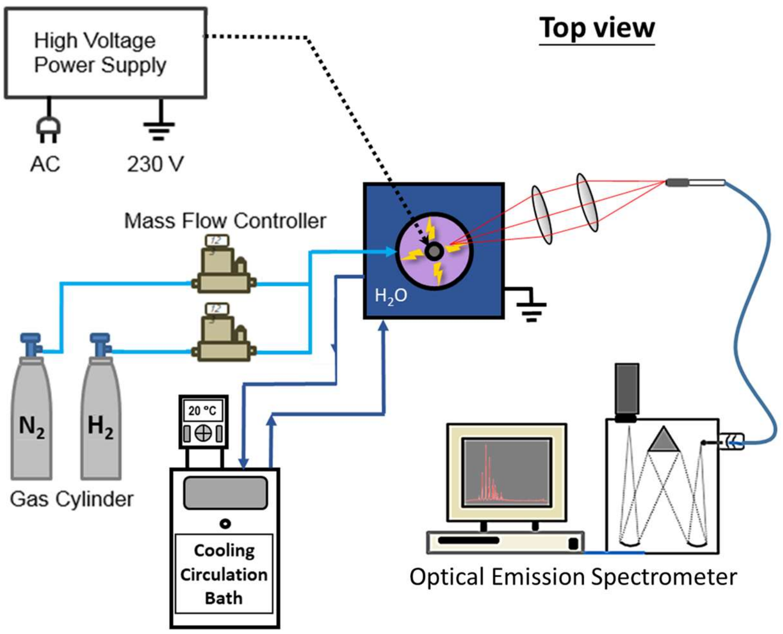

Figure S19. System schematic for the OES experiments.

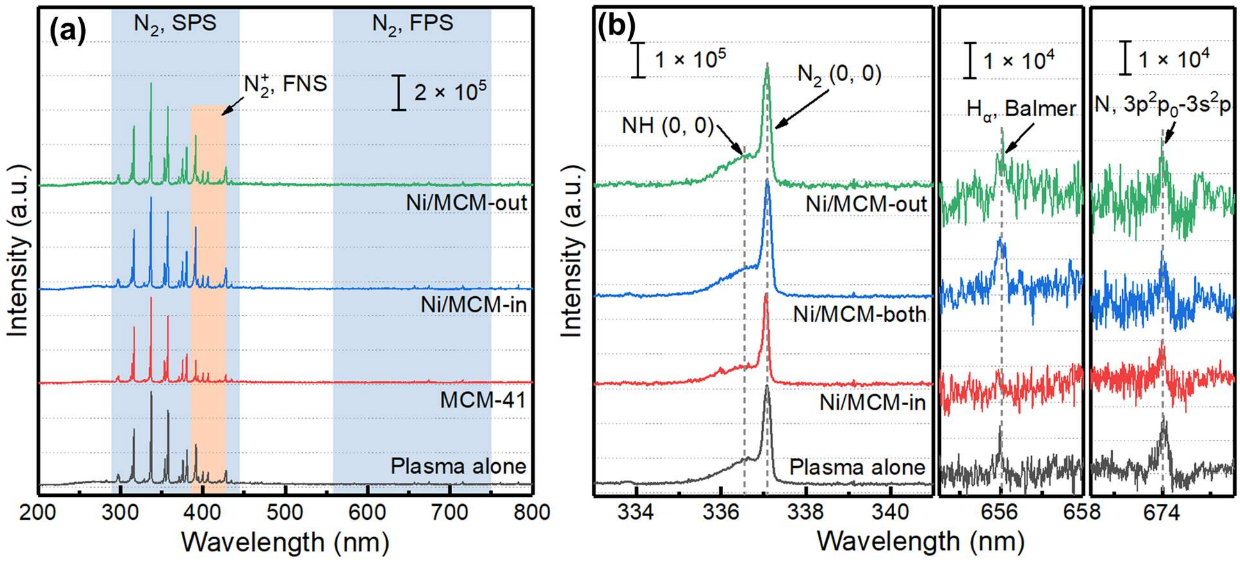

Figure S20. (a) Emission spectra for the plasma alone, and DBD systems with MCM-41, Ni/MCM-in and Ni/MCM-out (SEI = 30 kJ/L, exposure time = 600 ms, optical grating: 600 g/mm) (SPS = second positive system; FPS = first positive system; FNS = first negative system); (b) Amplified peaks for NH (0, 0), N_2 (0, 0), H_α (Balmer) and N radicals, respectively. (SEI = 30 kJ/L, exposure time = 30 s, optical grating: 2400 g/mm).

11. Calculation of the density of external Ni sites

The density of external Ni sites (μmol/m^2) is defined as the ratio of moles of external Ni sites per gram of catalysts (μmol/g), confirmed by H_2-TPR, to the external surface area of the catalysts (m^2/g), as defined by eq S10.

$$\text{External Ni site density } (\mu\text{mol/m}^2) = \frac{\text{Number of moles of external Ni sites } (\mu\text{mol/g})}{\text{External surface area } (\text{m}^2/\text{g})} \quad \text{(S10)}$$

Table S9. Quantitative results of external Ni site density of catalysts

	External Ni sites (μmol/g) [a]	External surface area (m^2/g) [b]	External Ni site density (μmol/m^2)
Ni/MCM-in	0	221	0
Ni/MCM-both	23.9	238	0.10
Ni/MCM-out	42.1	246	0.17
Ni/SiO_2	53.2	223	0.24

[a] Number of moles of external Ni sites are determined by the H_2 consumption of the α peak in H_2-TPR of the as-prepared catalysts; [b] External surface area is determined by BET surface area.

References

(1) Pérez, O. L.; Romeu, D.; Yacamán, M. J. The Relation between Dispersion and Particle Size on Supported Catalysts. *J. Catal.* **1983**, *79* (1), 240–241. https://doi.org/10.1016/0021-9517(83)90312-3.

(2) La-Salvia, N.; Lovón-Quintana, J. J.; Lovón, A. S. P.; Valença, G. P. Influence of Aluminum Addition in the Framework of MCM-41 Mesoporous Molecular Sieve Synthesized by Non-Hydrothermal Method in an Alkali-Free System. *Mater. Res.* **2017**, *20* (6), 1461–1469. https://doi.org/10.1590/1980-5373-MR-2016-1064.

(3) Wang, W.; Xue, K.; Pan, P.; Duan, J. Preparation of Bifunctional Core-Shell Structured Cu/TS-1@MCM-41 Molecular Sieves for Synthesis of Aniline by Benzene in One Step Amination. *Microporous Mesoporous Mater.* **2019**, *277* (November 2018), 163–170. https://doi.org/10.1016/j.micromeso.2018.10.038.

(4) Anderson, J. R. *Structure of Metallic Catalysts*; 1975.

(5) Tu, X.; Gallon, H. J.; Twigg, M. V; Gorry, P. A.; Whitehead, J. C. Dry Reforming of

Methane over a Ni/Al_2O_3 Catalyst in a Coaxial Dielectric Barrier Discharge Reactor. *J. Phys. D. Appl. Phys.* **2011**, *44* (27), 274007. https://doi.org/10.1088/0022-3727/44/27/274007.

(6) Wagner, H. E.; Brandenburg, R.; Kozlov, K. V.; Sonnenfeld, A.; Michel, P.; Behnke, J. F. The Barrier Discharge: Basic Properties and Applications to Surface Treatment. *User Model. User-adapt. Interact.* **2003**, *71* (3 SPEC.), 417–436. https://doi.org/10.1016/S0042-207X(02)00765-0.

(7) Kogelschatz, U. Dielectric-Barrier Discharges: Their History, Discharge Physics, and Industrial Applications. *Plasma Chem. Plasma Process.* **2003**, *23* (1), 1–46. https://doi.org/10.1023/A:1022470901385.

(8) Ramakers, M.; Michielsen, I.; Aerts, R.; Meynen, V.; Bogaerts, A. Effect of Argon or Helium on the CO2 Conversion in a Dielectric Barrier Discharge. *Plasma Process. Polym.* **2015**, *12*, 755–763. https://doi.org/10.1002/ppap.201400213.

(9) Rahmani, A.; Nikravech, M. Impact of Argon in Reforming of (CH4+ CO2) in Surface Dielectric Barrier Discharge Reactor to Produce Syngas and Liquid Fuels. *Plasma Chem. Plasma Process.* **2018**, *38* (3), 517–534. https://doi.org/10.1007/s11090-018-9886-2.

(10) Valdivia-Barrientos, R.; Pacheco-Sotelo, J.; Pacheco-Pacheco, M.; Benítez-Read, J. S.; López-Callejas, R. Analysis and Electrical Modelling of a Cylindrical DBD Configuration at Different Operating Frequencies. *Plasma Sources Sci. Technol.* **2006**, *15* (2), 237–245. https://doi.org/10.1088/0963-0252/15/2/008.

(11) Shah, J. R.; Gorky, F.; Lucero, J.; Carreon, M. A. M. L.; Carreon, M. A. M. L. Ammonia Synthesis via Atmospheric Plasma Catalysis: Zeolite 5A, a Case of Study. *Ind. Eng. Chem. Res.* **2020**, *59* (11), 5167–5176. https://doi.org/10.1021/acs.iecr.9b05220.

(12) Shah, J.; Wu, T.; Lucero, J.; Carreon, M. A. M. L.; Carreon, M. A. M. L. Nonthermal Plasma Synthesis of Ammonia over Ni-MOF-74. *ACS Sustain. Chem. Eng.* **2019**, *7* (1), 377–383. https://doi.org/10.1021/acssuschemeng.8b03705.

(13) Gorky, F.; Lucero, J. M.; Crawford, J. M.; Blake, B.; Carreon, M. A.; Carreon, M. L. Plasma-Induced Catalytic Conversion of Nitrogen and Hydrogen to Ammonia over Zeolitic Imidazolate Frameworks ZIF-8 and ZIF-67. *ACS Appl. Mater. Interfaces* **2021**, *13* (18), 21338–21348. https://doi.org/10.1021/acsami.1c03115.

(14) Gorky, F.; Guthrie, S. R.; Smoljan, C. S.; Crawford, J. M.; Carreon, M. A.; Carreon, M. L. Plasma Ammonia Synthesis over Mesoporous Silica SBA-15. *J. Phys. D. Appl. Phys.* **2021**, *54* (26). https://doi.org/10.1088/1361-6463/abefbc.

(15) Peng, P.; Cheng, Y.; Hatzenbeller, R.; Addy, M.; Zhou, N.; Schiappacasse, C.; Chen, D.; Zhang, Y.; Anderson, E.; Liu, Y.; et al. Ru-Based Multifunctional Mesoporous Catalyst for Low-Pressure and Non-Thermal Plasma Synthesis of Ammonia. *Int. J. Hydrogen Energy* **2017**, *42* (30), 19056–19066. https://doi.org/10.1016/j.ijhydene.2017.06.118.

(16) Li, S.; Shao, Y.; Chen, H.; Fan, X. Nonthermal Plasma Catalytic Ammonia Synthesis over a Ni Catalyst Supported on MgO/SBA-15. *Ind. Eng. Chem. Res.* **2022**, *61* (9), 3292–3302. https://doi.org/10.1021/acs.iecr.1c04968.

(17) Mizushima, T.; Matsumoto, K.; Ohkita, H.; Kakuta, N. Catalytic Effects of Metal-Loaded Membrane-like Alumina Tubes on Ammonia Synthesis in Atmospheric Pressure Plasma by Dielectric Barrier Discharge. *Plasma Chem. Plasma Process.* **2007**, *27* (1), 1–11. https://doi.org/10.1007/s11090-006-9034-2.

(18) Gómez-Ramŕez, A.; Cotrino, J.; Lambert, R. M.; González-Elipe, A. R. Efficient Synthesis of Ammonia from N2 and H2 Alone in a Ferroelectric Packed-Bed DBD Reactor. *Plasma Sources Sci. Technol.* **2015**, *24* (6). https://doi.org/10.1088/0963-0252/24/6/065011.

(19) Peng, P.; Li, Y.; Cheng, Y.; Deng, S.; Chen, P.; Ruan, R. Atmospheric Pressure Ammonia Synthesis Using Non-Thermal Plasma Assisted Catalysis. *Plasma Chem. Plasma Process.* **2016**, *36* (5), 1201–1210. https://doi.org/10.1007/s11090-016-9713-6.

(20) Gómez-Ramírez, A.; Montoro-Damas, A. M.; Cotrino, J.; Lambert, R. M.; González-Elipe, A. R. About the Enhancement of Chemical Yield during the Atmospheric Plasma Synthesis of Ammonia in a Ferroelectric Packed Bed Reactor. *Plasma Process. Polym.* **2017**, *14* (6), 1–8. https://doi.org/10.1002/ppap.201600081.

(21) Kim, H. H.; Teramoto, Y.; Ogata, A.; Takagi, H.; Nanba, T. Atmospheric-Pressure Nonthermal Plasma Synthesis of Ammonia over Ruthenium Catalysts. *Plasma Process. Polym.* **2017**, *14* (6), 1–9. https://doi.org/10.1002/ppap.201600157.

(22) Akay, G.; Zhang, K. Process Intensification in Ammonia Synthesis Using Novel Coassembled Supported Microporous Catalysts Promoted by Nonthermal Plasma. *Ind. Eng. Chem. Res.* **2017**, *56* (2), 457–468. https://doi.org/10.1021/acs.iecr.6b02053.

(23) Iwamoto, M.; Akiyama, M.; Aihara, K.; Deguchi, T. Ammonia Synthesis on Wool-Like Au, Pt, Pd, Ag, or Cu Electrode Catalysts in Nonthermal Atmospheric-Pressure Plasma of N_2 and H_2. *ACS Catal.* **2017**, *7* (1), 6924–6929. https://doi.org/10.1021/acscatal.7b01624.

(24) Mehta, P.; Barboun, P.; Herrera, F. A.; Kim, J.; Rumbach, P.; Go, D. B.; Hicks, J. C.; Schneider, W. F. Overcoming Ammonia Synthesis Scaling Relations with Plasma-Enabled

Catalysis. *Nat. Catal.* **2018**, *1* (April), 269–275. https://doi.org/10.1038/s41929-018-0045-1.

(25) Wang, Y.; Craven, M.; Yu, X.; Ding, J.; Bryant, P.; Huang, J.; Tu, X. Plasma-Enhanced Catalytic Synthesis of Ammonia over a Ni/Al_2O_3 Catalyst at near-Room Temperature: Insights into the Importance of the Catalyst Surface on the Reaction Mechanism. *ACS Catal.* **2019**, *9*, 10780–10793. https://doi.org/10.1021/acscatal.9b02538.

(26) Barboun, P.; Mehta, P.; Herrera, F. A.; Go, D. B.; Schneider, W. F.; Hicks, J. C. Distinguishing Plasma Contributions to Catalyst Performance in Plasma-Assisted Ammonia Synthesis. *ACS Sustain. Chem. Eng.* **2019**, *7* (9), 8621–8630. https://doi.org/10.1021/acssuschemeng.9b00406.

(27) Xie, Q.; Zhuge, S.; Song, X.; Lu, M.; Yu, F.; Ruan, R.; Nie, Y. Non-Thermal Atmospheric Plasma Synthesis of Ammonia in a DBD Reactor Packed with Various Catalysts. *J. Phys. D. Appl. Phys.* **2020**, *53* (6). https://doi.org/10.1088/1361-6463/ab57e5.

(28) Winter, L. R.; Ashford, B.; Hong, J.; Murphy, A. B.; Chen, J. G. Identifying Surface Reaction Intermediates in Plasma Catalytic Ammonia Synthesis. *ACS Catal.* **2020**, *10* (24), 14763–14774. https://doi.org/10.1021/acscatal.0c03166.

(29) Mehta, P.; Barboun, P. M.; Engelmann, Y.; Go, D. B.; Bogaerts, A.; Schneider, W. F.; Hicks, J. C. Plasma-Catalytic Ammonia Synthesis beyond the Equilibrium Limit. *ACS Catal.* **2020**, *10* (12), 6726–6734. https://doi.org/10.1021/acscatal.0c00684.

(30) Patil, B. S.; Cherkasov, N.; Srinath, N. V.; Lang, J.; Ibhadon, A. O.; Wang, Q.; Hessel, V. The Role of Heterogeneous Catalysts in the Plasma-Catalytic Ammonia Synthesis. *Catal. Today* **2021**, *362* (November 2019), 2–10. https://doi.org/10.1016/j.cattod.2020.06.074.

(31) Zhu, X.; Hu, X.; Wu, X.; Cai, Y.; Zhang, H.; Tu, X. Ammonia Synthesis over γ-Al2O3 Pellets in a Packed-Bed Dielectric Barrier Discharge Reactor. *J. Phys. D. Appl. Phys.* **2020**, *53* (16). https://doi.org/10.1088/1361-6463/ab6cd1.

(32) Patil, B. S.; Van Kaathoven, A. S. R.; Peeters, F. J. J.; Cherkasov, N.; Lang, J.; Wang, Q.; Hessel, V. Deciphering the Synergy between Plasma and Catalyst Support for Ammonia Synthesis in a Packed Dielectric Barrier Discharge Reactor. *J. Phys. D. Appl. Phys.* **2020**, *53* (14), ab6a36. https://doi.org/10.1088/1361-6463/ab6a36.

(33) Li, S.; van Raak, T.; Gallucci, F. Investigating the Operation Parameters for Ammonia Synthesis in Dielectric Barrier Discharge Reactors. *J. Phys. D. Appl. Phys.* **2020**, *53* (1), 014008. https://doi.org/10.1088/1361-6463/ab4b37.

(34) Iwamoto, M.; Horikoshi, M.; Hashimoto, R.; Shimano, K.; Sawaguchi, T.; Teduka, H.; Matsukata, M. Higher Activity of Ni/γ-Al2O3 over Fe/γ-Al2O3 and Ru/γ- Al2O3 for Catalytic Ammonia Synthesis in Nonthermal Atmospheric-Pressure Plasma of N2 and H2. *Catalysts* **2020**, *10* (5), 590. https://doi.org/10.3390/catal10050590.

(35) Rouwenhorst, K. H. R.; Burbach, H. G. B.; Vogel, D. W.; Núñez Paulí, J.; Geerdink, B.; Lefferts, L. Plasma-Catalytic Ammonia Synthesis beyond Thermal Equilibrium on Ru-Based Catalysts in Non-Thermal Plasma. *Catal. Sci. Technol.* **2021**, *11* (8), 2834–2843. https://doi.org/10.1039/d0cy02189j.

(36) Hu, X.; Zhu, X.; Wu, X.; Cai, Y.; Tu, X. Plasma-Enhanced NH3 Synthesis over Activated Carbon-Based Catalysts: Effect of Active Metal Phase. *Plasma Process. Polym.* **2020**, *17* (12), 1–9. https://doi.org/10.1002/ppap.202000072.

(37) Mao, C.; Li, H.; Gu, H.; Wang, J.; Zou, Y.; Qi, G.; Xu, J.; Deng, F.; Shen, W.; Li, J.; et al. Beyond the Thermal Equilibrium Limit of Ammonia Synthesis with Dual Temperature Zone Catalyst Powered by Solar Light. *Chem* **2019**, *5* (10), 2702–2717. https://doi.org/10.1016/j.chempr.2019.07.021.

(38) Sayan, Ş.; Kantcheva, M.; Suzer, S.; Uner, D. O. FTIR Characterization of Ru/SiO2 Catalyst for Ammonia Synthesis. *J. Mol. Struct.* **1999**, *480–481*, 241–245. https://doi.org/10.1016/S0022-2860(98)00812-6.

(39) Ramis, G.; Larrubia, M. A. An FT-IR Study of the Adsorption and Oxidation of N-Containing Compounds over Fe2O3/Al2O3 SCR Catalysts. *J. Mol. Catal. A Chem.* **2004**, *215* (1–2), 161–167. https://doi.org/10.1016/j.molcata.2004.01.016.

(40) Yao, Y.; Zhu, S.; Wang, H.; Li, H.; Shao, M. A Spectroscopic Study on the Nitrogen Electrochemical Reduction Reaction on Gold and Platinum Surfaces. *J. Am. Chem. Soc.* **2018**, *140* (4), 1496–1501. https://doi.org/10.1021/jacs.7b12101.

(41) Ramis, G.; Yi, L.; Busca, G.; Turco, M.; Kotur, E.; Willey, R. J. Adsorption, Activation, and Oxidation of Ammonia over SCR Catalysts. *J. Catal.* **1995**, *157*, 523–535.

(42) Yao, Y.; Wang, H.; Yuan, X. Z.; Li, H.; Shao, M. Electrochemical Nitrogen Reduction Reaction on Ruthenium. *ACS Energy Lett.* **2019**, *4* (6), 1336–1341. https://doi.org/10.1021/acsenergylett.9b00699.

(43) O'Donoghue, M. B.; Davis, W. M.; Schrock, R. R. Derivatization of Dinitrogen by Molybdenum in Triamidoamine Complexes. *Inorg. Chem.* **1998**, *37* (20), 5149–5158. https://doi.org/10.1021/ic9803513.

(44) Yandulov, D. V.; Schrock, R. R.; Rheingold, A. L.; Ceccarelli, C.; Davis, W. M. Synthesis and Reactions of Molybdenum Triamidoamine Complexes Containing Hexaisopropylterphenyl Substituents. *Inorg. Chem.* **2003**, *42* (3), 796–813. https://doi.org/10.1021/ic020505l.

(45) Allouche, A. Software News and Updates Gabedit — A Graphical User Interface for Computational Chemistry Softwares. *J. Comput. Chem.* **2012**, *32*, 174–182. https://doi.org/10.1002/jcc.

(46) Jia, H. P.; Quadrelli, E. A. Mechanistic Aspects of Dinitrogen Cleavage and Hydrogenation to Produce Ammonia in Catalysis and Organometallic Chemistry: Relevance of Metal Hydride Bonds and Dihydrogen. *Chem. Soc. Rev.* **2014**, *43* (2), 547–564. https://doi.org/10.1039/c3cs60206k.
